# Supplementary material for: Pluripotent, germ cell competent adult stem cells underlie cnidarian regenerative ability and clonal growth
Source: Curr Biol. 2023 May 22;33(10):1883–1892.e3. doi: 10.1016/j.cub.2023.03.039 (PMC10234598; doi:10.1016/j.cub.2023.03.039)
Supplement: Document S1. Figures S1–S6 [file mmc1.pdf]

Current Biology, Volume *33*

Supplemental Information

Pluripotent, germ cell competent adult stem cells underlie cnidarian regenerative ability and clonal growth

Áine Varley, Helen R. Horkan, Emma T. McMahon, Gabriel Krasovec, and Uri Frank

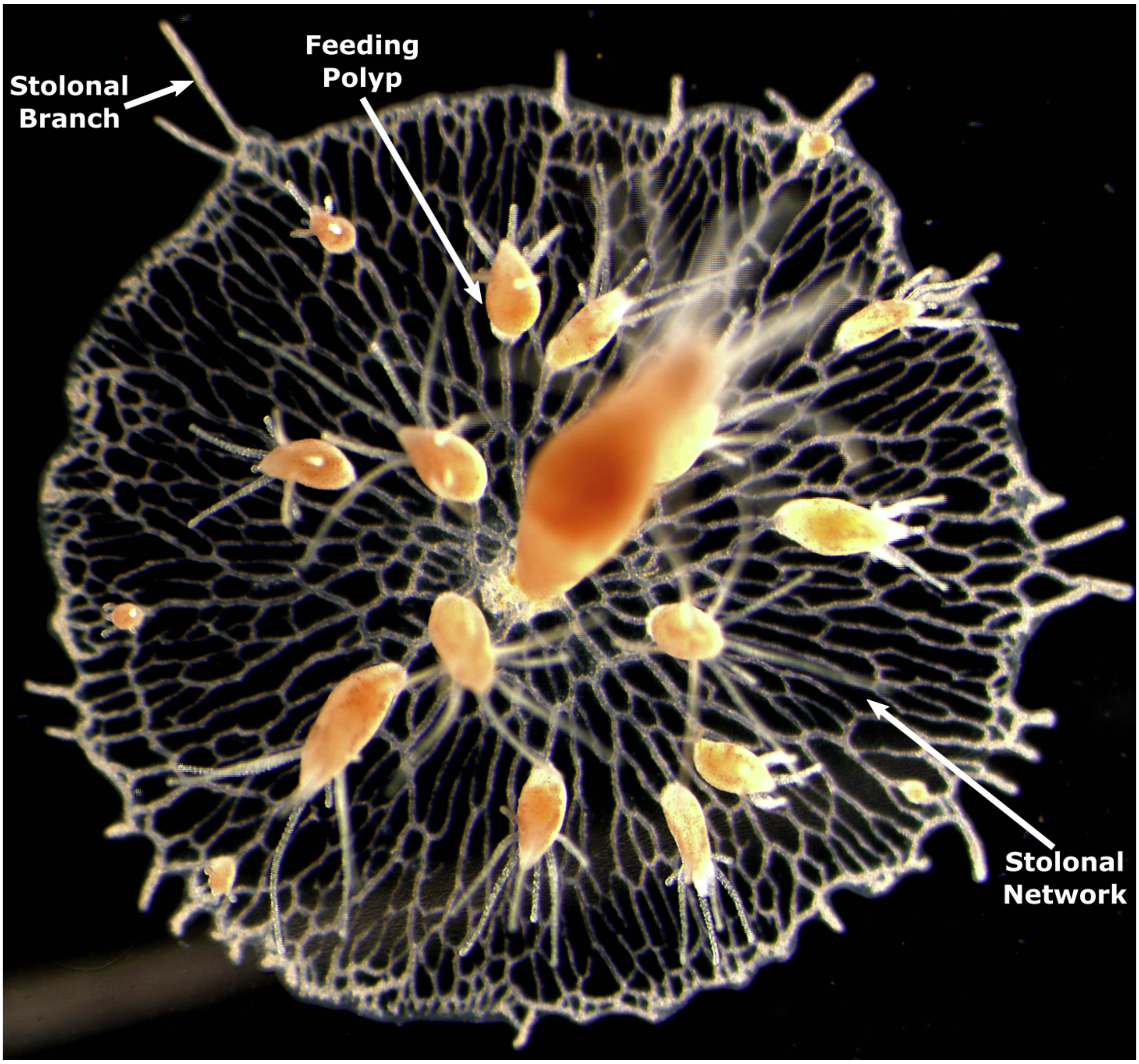

Figure S1. **Feeding polyps and stolonal network of a young colony. Related to Figure 1.**

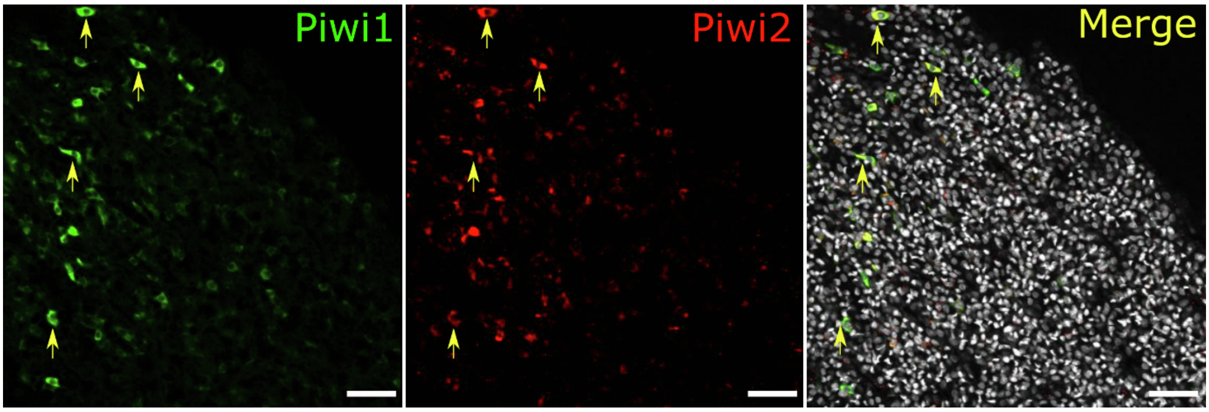

Figure S2. **Piwi1 and Piwi2 double labeling of i-cells. Related to Figure 2.**

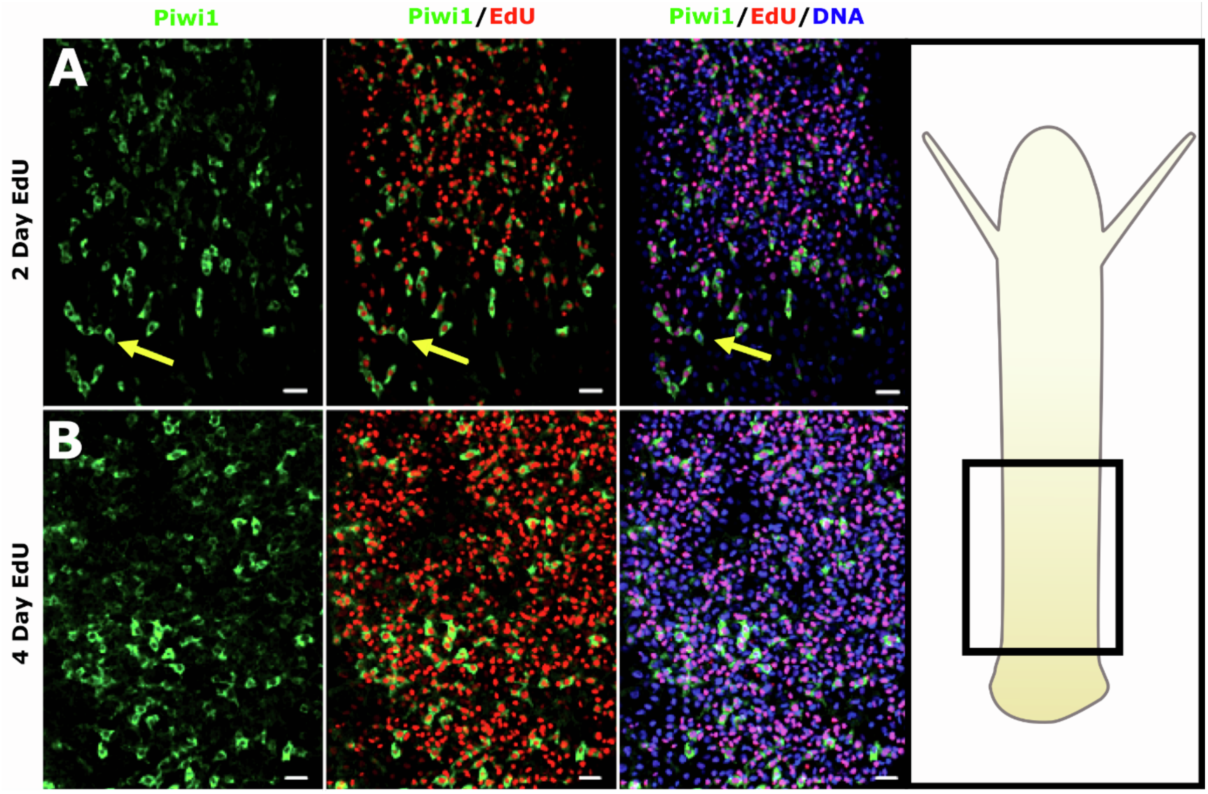

Figure S3. **Fast cycling i-cells in polyps. Related to Figure 2.** (A) EdU incorporation in feeding polyps after 2 days. (B) EdU incorporation after 4 days. Nearly all i-cells have gone through S-phase and are EdU^{+}. Arrow indicates EdU negative i-cell. Scale bars 20 μm.

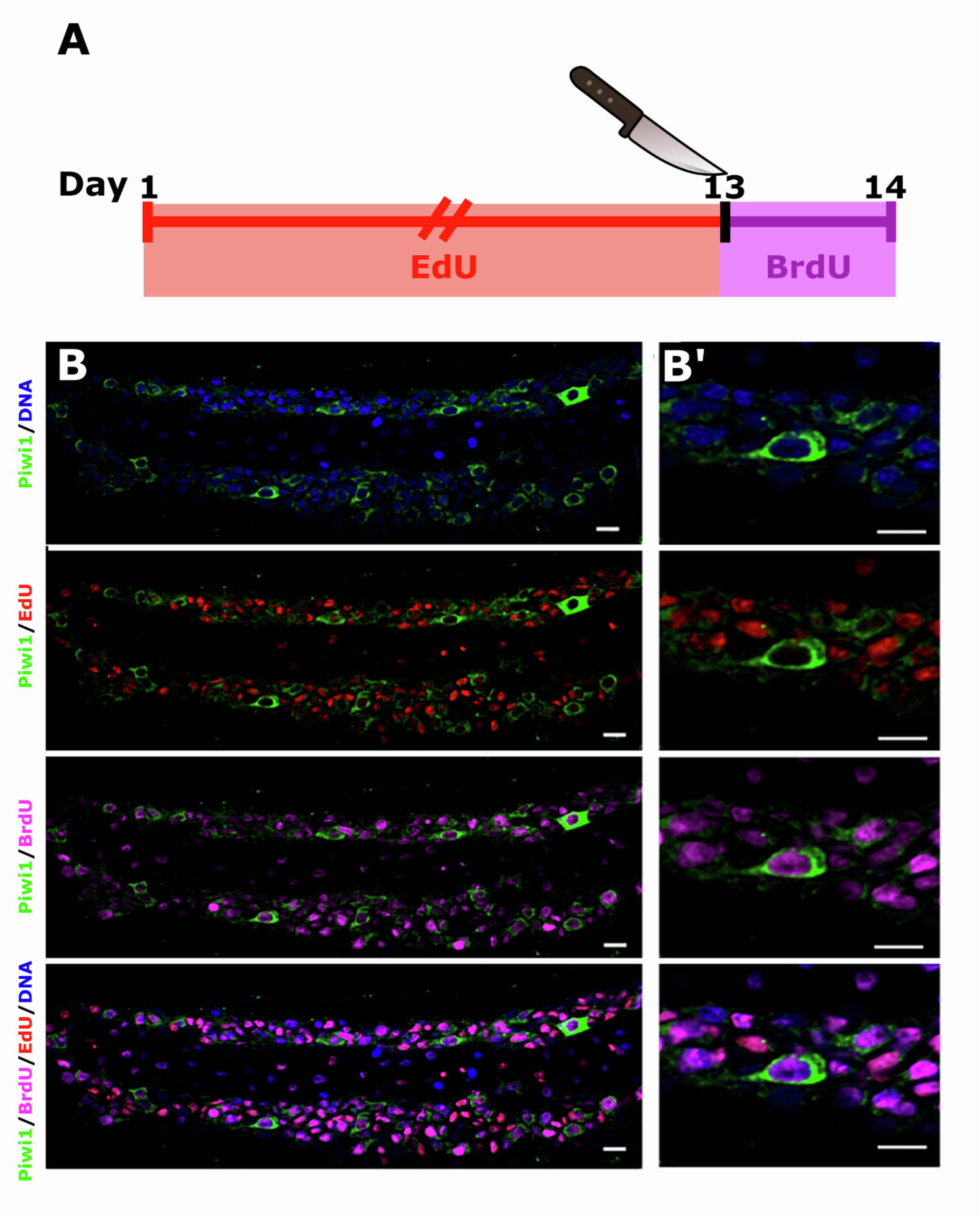

Figure S4. **Slow cycling i-cells in stolons. Related to Figure 2.** (A) Animals were incubated in EdU for 12 days, injured, and incubated in BrdU for 24 hours. (B) i-cells that had been quiescent for at least 12 days but reentered the cell cycle following injury are shown. (B') Higher magnification of a single i-cell. Scale bars 10 µm.

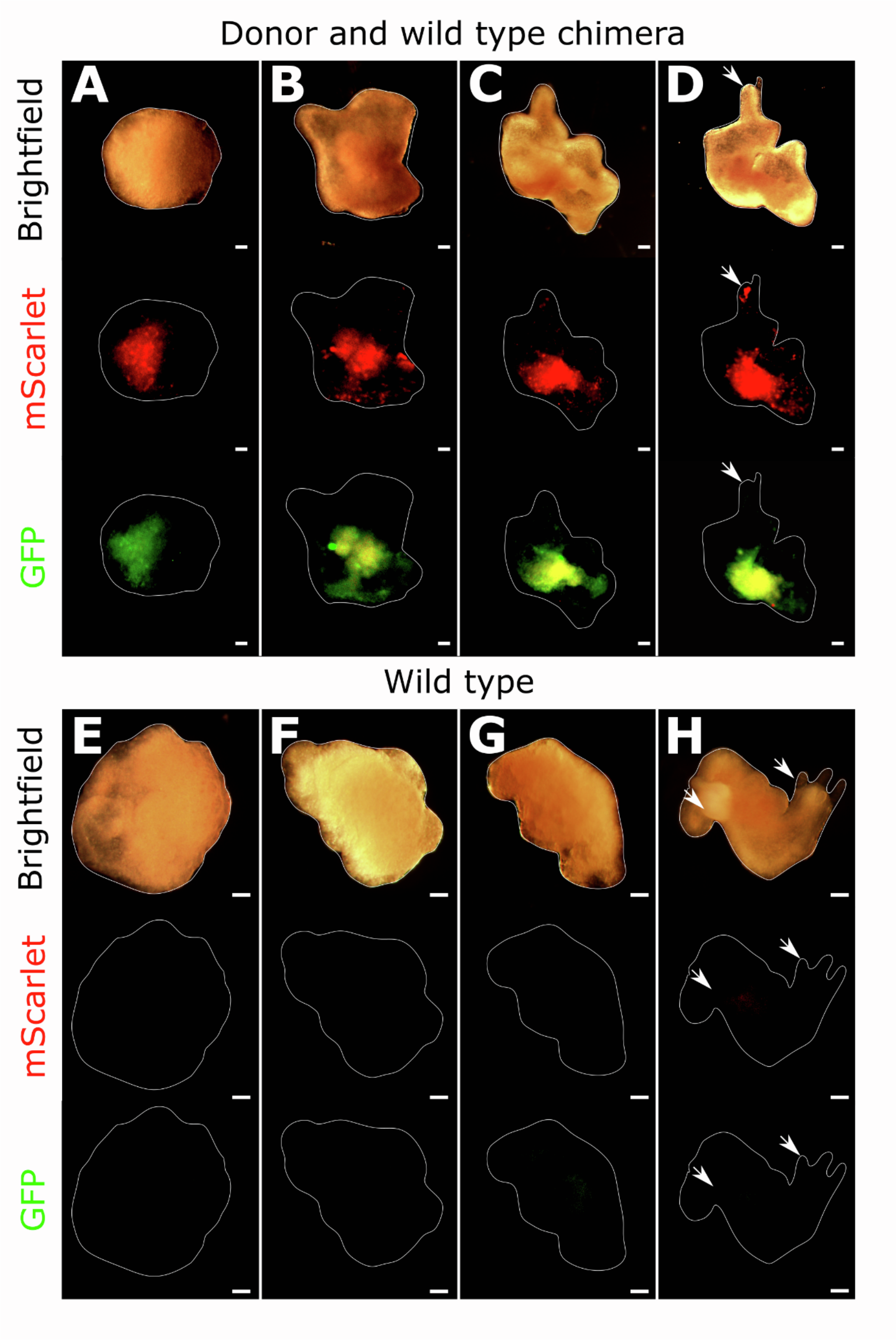

Figure S5. Live imaging of reaggregated dissociated cells. Related to Figure 3. (A-D) Mixed cell aggregate composed of dissociated 291-10 and 106 polyps. (E-H) Wild type (291-10) aggregates. (A&E) Day 1. (B&F) Day 3 (C&G) Day 5. (D&H) Day 7. Arrows denote regenerating polyps. Scale bars 20 µm.

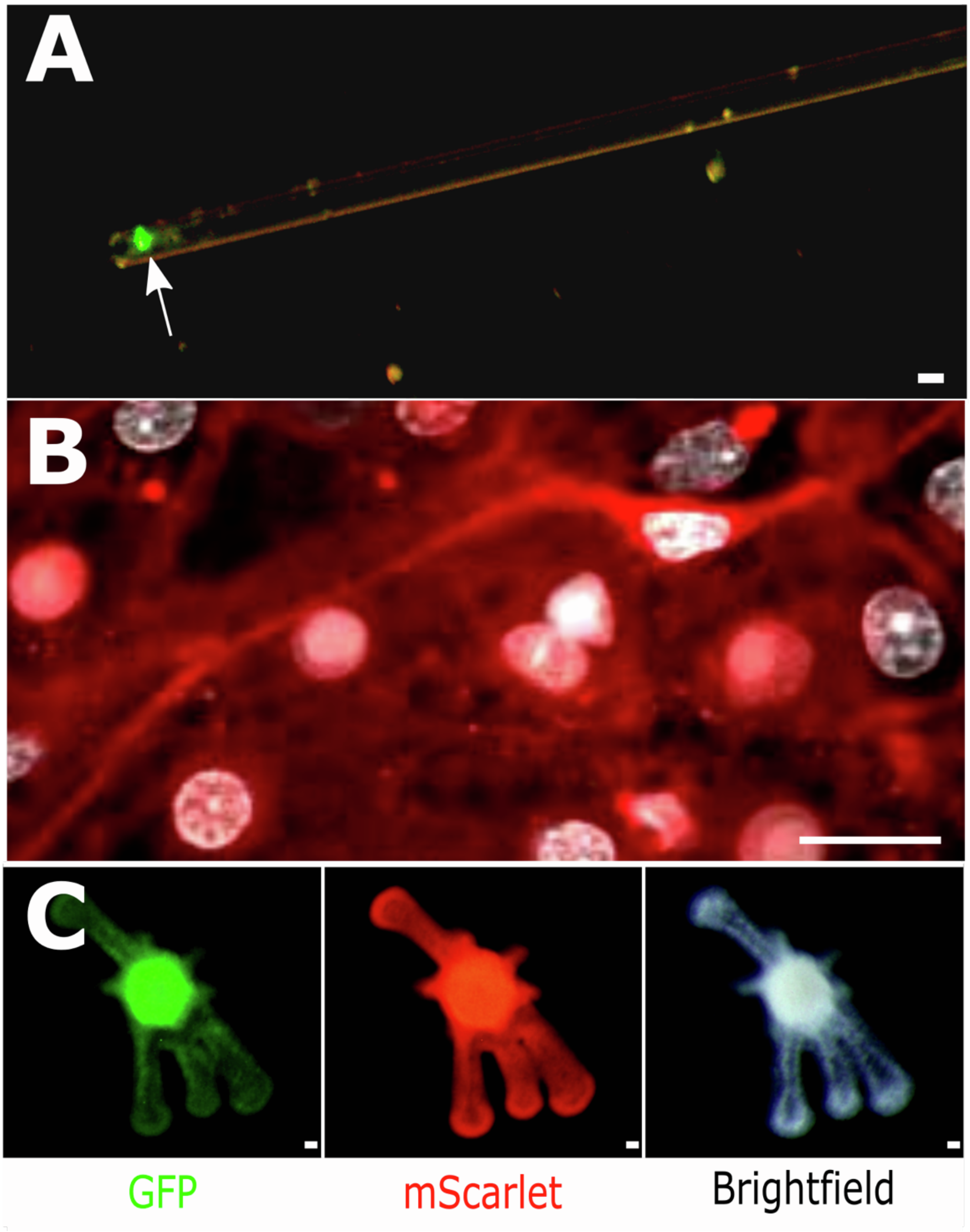

Figure S6. **Isolated transgenic i-cell, donor-derived neuron, and second-generation transgenic colony. Related to Figure 4.** (A) single transgenic i-cell, aspirated in a micropipette from a dissociated 106 polyp. (B) Live image of mScarlet-positive, donor-derived neuron embedded in a chimeric recipient's tissue. (DNA is shown in grey. (C) Young transgenic colony, derived from a single transplanted i-cell. Scale bars 10 µm in A and 20 µm in B & C.
