# Supplementary material for: Pluripotent, germ cell competent adult stem cells underlie cnidarian regenerative ability and clonal growth
Source: Curr Biol. 2023 May 22;33(10):1883–1892.e3. doi: 10.1016/j.cub.2023.03.039 (PMC10234598; doi:10.1016/j.cub.2023.03.039)
Supplement: Document S2. Article plus supplemental information [file mmc3.pdf]

Current Biology

Article

Pluripotent, germ cell competent adult stem cells underlie cnidarian regenerative ability and clonal growth

Graphical abstract

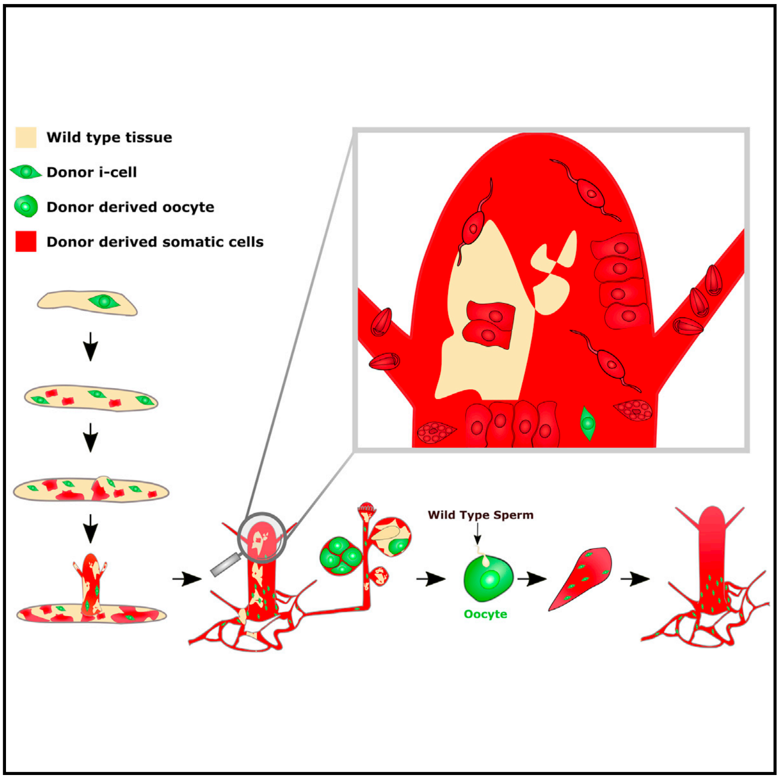

Highlights

- Adult stem cells in the cnidarian *Hydractinia*—known as i-cells—are pluripotent
- i-cells are marked by high levels of Piwi1, Piwi2, and Soxb1 expression
- The i-cell population includes continuously self-renewing and slow-cycling cells
- A single i-cell can differentiate into all somatic lineages and germ cells

Authors

Áine Varley, Helen R. Horkan, Emma T. McMahon, Gabriel Krasovec, Uri Frank

Correspondence

uri.frank@universityofgalway.ie

In brief

Cnidarians (corals and jellyfish) possess high regenerative abilities and do not succumb to age. These traits depend on a population of adult stem cells, known as i-cells. Varley et al. find that i-cells in the cnidarian *Hydractinia* are pluripotent. A single transplanted allogeneic i-cell can replace all cells of the host, including its germ cells.

Varley et al., 2023, Current Biology *33*, 1883–1892
May 22, 2023 © 2023 The Author(s). Published by Elsevier Inc.
https://doi.org/10.1016/j.cub.2023.03.039

CellPress

Current Biology

CellPress
OPEN ACCESS

Article

Pluripotent, germ cell competent adult stem cells underlie cnidarian regenerative ability and clonal growth

Áine Varley,[1] Helen R. Horkan,[1] Emma T. McMahon,[1] Gabriel Krasovec,[1] and Uri Frank[1,2,3,*]
[1]Centre for Chromosome Biology, School of Biological and Chemical Sciences, University of Galway, Galway H91W2TY, Ireland
[2]Twitter: @thecocodium
[3]Lead contact
*Correspondence: uri.frank@universityofgalway.ie
https://doi.org/10.1016/j.cub.2023.03.039

SUMMARY

In most animals, pluripotency is irreversibly lost post gastrulation. By this stage, all embryonic cells have already committed either to one of the somatic lineages (ectoderm, endoderm, or mesoderm) or to the germline. The lack of pluripotent cells in adult life may be linked to organismal aging. Cnidarians (corals and jellyfish) are an early branch of animals that do not succumb to age, but the developmental potential of their adult stem cells remains unclear. Here, we show that adult stem cells in the cnidarian *Hydractinia symbiolongicarpus* (known as i-cells) are pluripotent. We transplanted single i-cells from transgenic fluorescent donors to wild-type recipients and followed them *in vivo* in the translucent animals. Single engrafted i-cells self-renewed and contributed to all somatic lineages and gamete production, co-existing with and eventually displacing the allogeneic recipient's cells. Hence, a fully functional, sexually competent individual can derive from a single adult i-cell. Pluripotent i-cells enable regenerative, plant-like clonal growth in these animals.

INTRODUCTION

Pluripotency, the ability of a cell to differentiate into all somatic lineages and germ cells,[1] is a state that—in most animals—is restricted to early, pre-gastrulation embryos. *In vitro*, mammalian pluripotent cells can self-renew indefinitely while maintaining their plasticity.[2] Post gastrulation and throughout adulthood, most animals renew their tissues and regenerate using lineage-restricted stem cells, collectively known as tissue stem cells.[3,4] The limited ability of tissue stem cells to self-renew causes most animals to decline over time and eventually die.

Known exceptions to this rule are clonal invertebrates such as cnidarians and planarians. Some of these animals show little or no evidence for aging and can regenerate whole bodies, including germ cells, from small tissue fragments.[5–8] Well-studied clonal animals possess adult stem cells, but their properties at single-cell resolution have only been studied in two animals—one planarian and one cnidarian. Single adult stem cells in the planarian *Schmidtea mediterranea* (known as neoblasts) were shown to contribute to all somatic lineages. However, their germ cell competence remains uncertain, given that single-cell experiments were only carried out with an asexual strain of this species.[9,10] By contrast, stem cells in the cnidarian *Hydra magnipapillata* (known as i-cells) can give rise to cells of the neuroglandular lineage and to germ cells, but apparently not to the two epithelial layers of the body wall; the latter constitute distinct, self-renewing lineages.[11–14]

Both *Schmidtea* and *Hydra* are clonal yet solitary animals. Here, we addressed the developmental potential of i-cells in the clonal, colony-forming cnidarian *Hydractinia symbiolongicarpus* (Figures 1 and S1). Using single i-cell transplantation from transgenic fluorescent donors to wild-type recipients, we find that these cells are pluripotent. Under physiological conditions, a single i-cell can differentiate into all somatic cell types and into gametes. The presence of embryonic-like, pluripotent cells throughout adult life facilitates their ability to grow indefinitely in a plant-like fashion.

RESULTS

i-cells and their proliferative activity

Hydractinia i-cells first appear lodged in the interstitial spaces of the embryo's endoderm, like in other hydrozoans.[15] i-cells are marked by *Piwi1*, *Piwi2*, and *Soxb1* expression (Figure S2).[16] After metamorphosis of the swimming larva, the animal enters a sedentary life, attached to the substratum. It forms colonies that are composed of modular, clonal units called polyps that are interconnected by a network of gastrovascular tubes known as stolons (Figure S1).[17] *Hydractinia* colonies grow indefinitely in a plant-like fashion by elongating their stolons and budding new polyps, showing no signs of age-related deterioration.[18]

The cell cycle of i-cells lacks a pronounced G1 phase,[16] but whether these cells continuously cycle is unknown. We found that animals incubated in EdU for 4 days had nearly 100% of their

This is an open access article under the CC BY license (http://creativecommons.org/licenses/by/4.0/).

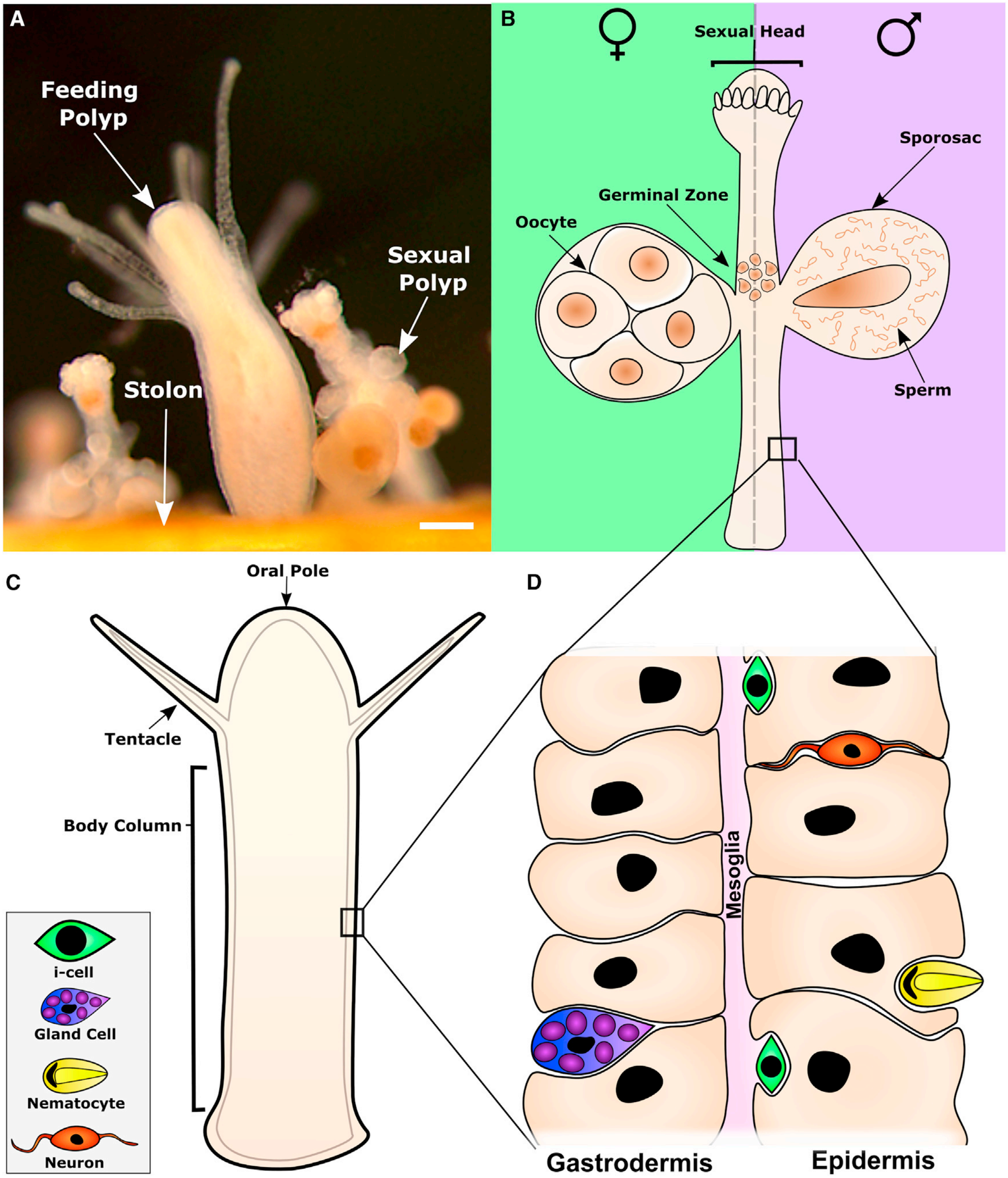

Figure 1. *Hydractinia* colony and body wall structure

(A) A colony is composed of feeding and sexual polyps and the stolonal tissue that interconnects them.

(B) Sexual polyps are structured as a cylindrical body column with a head equipped with rudimentary tentacles. i-cells commit to germ cell fate in the germinal zone. They then migrate and colonize the gamete containers, known as sporosacs.

(C) Feeding polyps are structured as a cylindrical body column with a head and tentacles, used to catch prey, around the mouth opening.

(D) The body walls of feeding and sexual polyps are composed of two epitheliomuscular layers, sandwiching a basement membrane known as the mesoglea. Other cell types, such as neurons, nematocytes, gland cells, and i-cells, are lodged in the interstitial spaces between epithelial cells. Scale bars, 40 μm.

See also Figure S1.

polyp i-cells labeled (Figure S3), except for a few unlabeled i-cells around the polyp-stolon interface. By contrast, many stolonal i-cells remained EdU free even after 12 days of EdU incubation (Figure S4). To investigate whether these non-cycling i-cells can re-enter the cell cycle, we injured the animals at day 12 of EdU incubation to induce a regenerative response, exposed them to bromodeoxyuridine (BrdU) for 24 h, fixed them, and visualized the EdU, BrdU, and Piwi1 protein (an i-cell marker). We

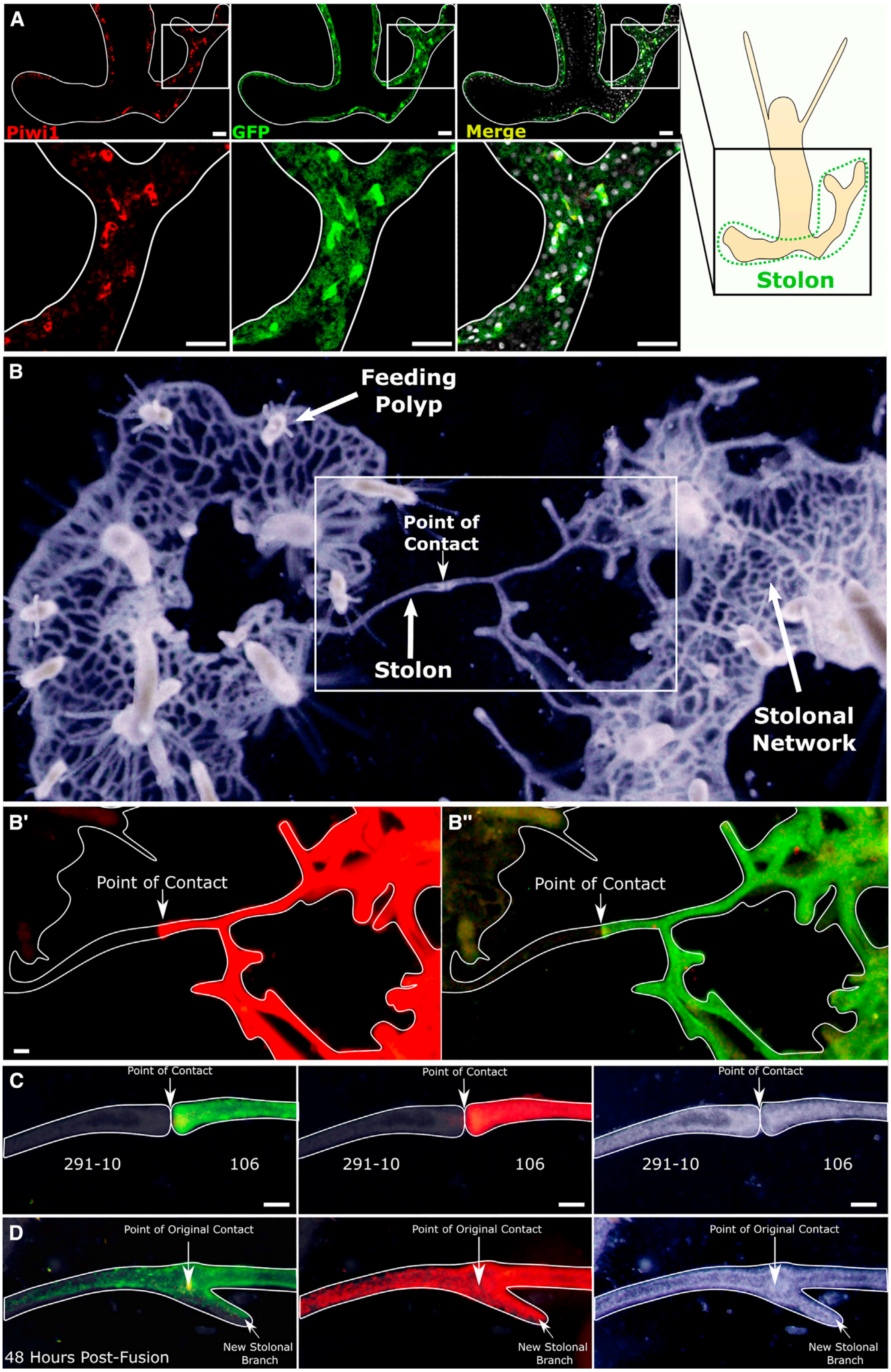

(legend on next page)

found many i-cells that were only BrdU positive (Figure S4). These cells had been quiescent for at least 12 days but reentered the cell cycle following injury. We concluded that, although most i-cells continuously cycle, a sub-population that is more common in stolons is slow cycling. Because isolated polyps are well able to regenerate stolons,[19] there is no evidence to suggest that polyp i-cells are distinct from their stolonal counterparts except that more stolonal i-cells appear to be slow cycling. This is probably because polyps must continuously renew their stinging cells, which are depleted each time the animal catches prey. The mid parts of stolons, where slow-cycling i-cells reside, do not grow.

The developmental potential of a single i-cell

At the population level, it has been shown that i-cells proliferate, migrate, and differentiate to replace disposable somatic cells and also generate gametes.[19–24] However, it remains unclear whether i-cells constitute several distinct, lineage-restricted stem cells or a pluripotent population. To address this question, we aimed to transplant a single, transgenic fluorescent i-cell into a wild-type recipient. As an i-cell donor, we used a female animal (clone 106) that carries two fluorescent reporter transgenes: one is a *Piwi1*-driven GFP; this reporter is only active in i-cells (Figure 2A) and germ cells but is downregulated following differentiation to somatic cells.[24] The other transgene is a β-tubulin-driven mScarlet; this transgene is expressed by all differentiated cells but suppressed in i-cells.[24] Hence, i-cells in this animal are readily identifiable, being only bright green and not red fluorescent. i-cell progeny and terminally differentiated cells are bright red and dim green because of the long half-life of GFP. Early germ cells express Piwi1::GFP and, as they mature, also β-tubulin:: mScarlet. They are anatomically restricted to sexual polyps.[24] Our rationale was to transplant a single, green-only i-cell into a wild-type host. Products of self-renewal should remain green only, whereas all their differentiating/differentiated progeny should turn red and their green GFP fluorescence gradually dissipate. As recipient host, we selected the wild-type male clone 291-10. The two animals are genetically histocompatible, accepting tissue grafts from each other to generate stable chimeras. The opposite sexes of donor and recipient provided an additional genetic marker to distinguish between donor and host gametes, in addition to donor fluorescence. This was possible because sex in *Hydractinia* is genetically determined by an XY system[25] and gamete production depends on the genetic sex of their i-cell progenitors rather than on the somatic gonadal environment. Therefore, male/female chimeras can contain eggs and sperm in the very same gonad.[21,26] *Hydractinia*'s unlimited clonal growth allowed us to generate multiple genetically identical copies of both animals, facilitating biological repeat experiments in the same genetic background.

We first attempted to generate allogeneic mixed cell aggregates, which can regenerate a fully functional individual. Donor and recipient polyps were dissociated into a single-cell suspension by incubating them in calcium- and magnesium-free seawater. Dissociated cells were reaggregated by centrifugation. The resulting cell pellet regenerated an intact animal by day 5 post aggregation. Adding many donor cells to wild-type recipient cell suspension resulted in successful engraftment and chimera establishment (Figure S5). However, when lowering the donor cell numbers to few or single i-cells, all our attempts to recover the fluorescent donor cells from the regenerated polyps failed, suggesting that engraftment under these conditions is highly ineffective. Indeed, on average, a cell pellet made of 25 dissociated polyps gave rise to a single polyp, showing that most cells are lost during dissociation and reaggregation. Gamma-irradiating wild-type animals at 50 Gy before dissociation had not resulted in successful engraftment of single donor-i-cells.

We then attempted to inject fluorescent i-cells into intact hosts. For this, we dissociated donor animals under a fluorescence stereomicroscope and picked up a single, green-only i-cell using a microinjector (Figure S6A). We then injected the cells into wild-type animals at different life stages. However, after more than 50 injections, none of the injected i-cells could be found in the hosts' tissue 24 h later, showing that engraftment of injected i-cells is ineffectual as well.

Poor engraftment of i-cells could have resulted from the physical stress associated with tissue dissociation and mechanical handling. To transplant i-cells from a donor to a recipient without exposing them to unnecessary stress, we used whole tissue grafts. In their natural habitat, *Hydractinia* colonies grow on the surface of hermit crab shells. If a single shell is colonized by more than one larva, post metamorphosis, when the resulting polyps grow clonally, extending stolons of two allogeneic colonies may come into contact. The outcomes of these encounters depend genetically on sharing alleles at the highly polymorphic allorecognition complex. Colonies that share at least one allele of the complex can fuse and form chimeras that exchange migratory i-cells,[23] whereas no shared alleles results in an aggressive rejection.[27,28]

We positioned wild type 291-10 colonies close to a growing stolon of transgenic fluorescent donors (clone 106) on glass slides. Stolons of both histocompatible colonies established contact, fused, and fluorescent i-cells were observed migrating from the female 106 colonies to the male 291-10 tissue (Figures 2B–2D). We attempted to isolate a section of 291-10 stolon with only one, green donor i-cell. However, immigrating

Figure 2. Allogeneic stolonal fusion and chimera establishment by 106 and 291-10 colonies
(A) Co-localization of Piwi1 (red) and GFP (green) in a transgenic reporter polyp and its stolon.
(B) The stolonal network and polyps of two colonies growing to direct stolonal contact. The colony on the left is 291-10, and the one on the right is 106. Boxed area (B′) shows the mScarlet channel and (B″) the GFP.
(C) Higher magnification of the point of contact.
(D) The same parabiosis, 2 days post fusion. Note numerous i-cells (green) and differentiated cells (red) migrating from the 106 stolon into the 291-10 clone's tissue.
(B)–(D) are live images. Scale bars, 40 μm.
See also Figures S2–S4.

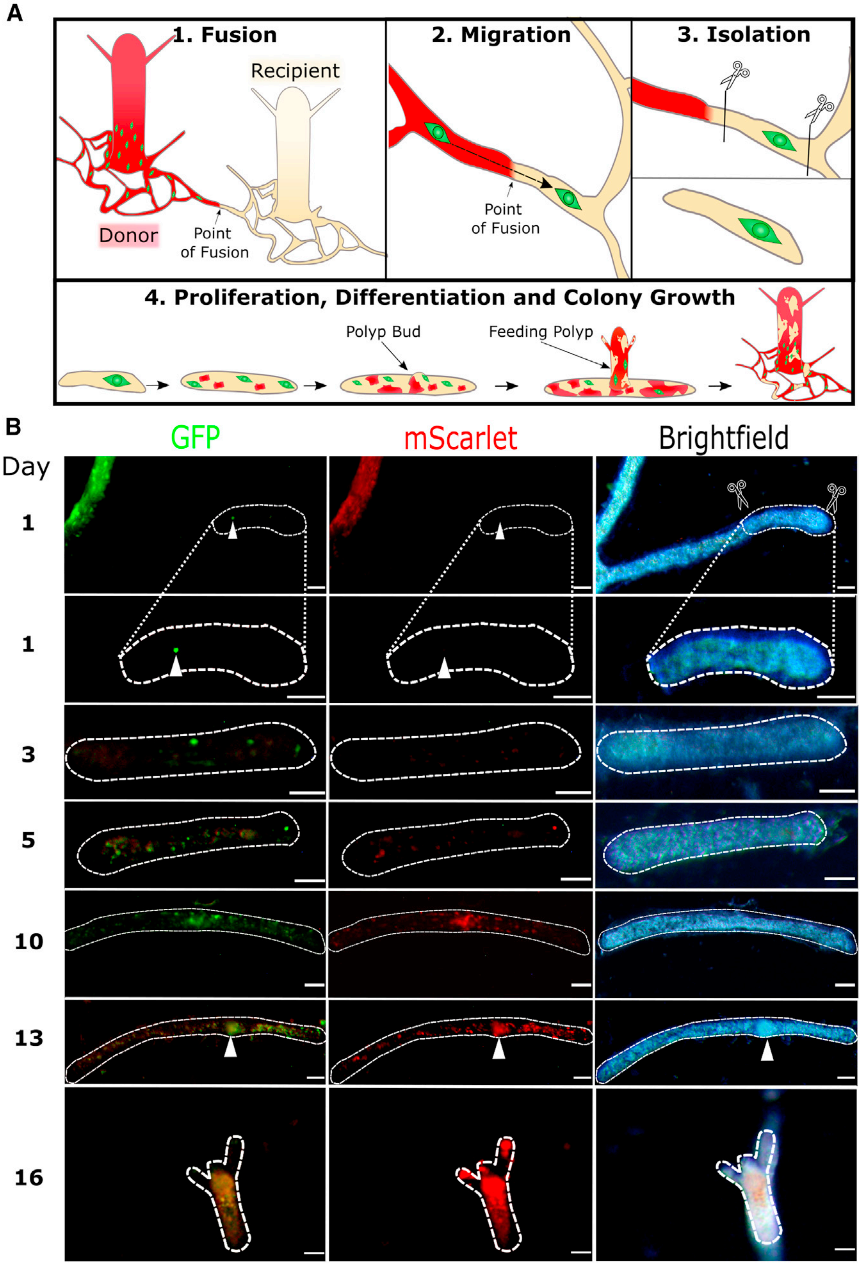

Figure 3. Single i-cell transplantation by parabiosis

(A) A schematic of colony grafting. A wild-type colony is positioned in close proximity to a transgenic fluorescent colony. Allogeneic stolonal fusion allows migratory i-cells to move from one colony to the other without leaving their niche. A section of wild-type stolon with only one donor fluorescent i-cells is isolated by removing the donor and recipient tissues.

(B) Live imaging of the timeline of a single-cell graft. First chimeric polyp bud was visible on day 13 (arrowhead). Fully developed polyp, capable of feeding, was established by day 16. Donor cells are green (i-cells) and red (differentiated cells). A total of five single-cell grafts were generated, and all revealed the same outcome. Scale bars, 40 μm.

See also Figure S5.

i-cells (green) and progeny (red) were too numerous (Figure 2D). Therefore, we generated chimeras in which most cells were wild type 291-10-derived, with only a few fluorescent cells from the 106 clone. We then grafted 291-10 colonies onto the chimeras. Because the number of donor fluorescent cells in the chimera was low, green immigrating i-cells were rare, allowing us to isolate sections of 291-10 wild-type stolons with only one green fluorescent (106-derived) i-cell by cutting away the rest of the donor and recipient tissues (Figure 3A). The size of these isolated stolonal pieces, which included no polyps, was less than 300 μm long, 40 μm wide, and 15 μm thick. This was small enough to exclude the presence of additional donor i-cells or progeny using fluorescence microscopy (Figure 3B) but contained sufficient cells to regenerate a whole new animal.

Out of over 120 established parabioses, in only five cases were we able to isolate a small piece of stolon with only one donor i-cell (green only) and no donor progeny (green and red, or red only). This is because cell migration patterns are unpredictable, with cells changing their migratory behavior within hours.

We allowed the five single-cell grafts to bud new polyps, feed, and become established colonies while taking daily observations under the fluorescence stereomicroscope. In each one of these chimeric animals, the single, green-only i-cell proliferated and gave rise both to new, green-only i-cells and to differentiated progeny that expressed red mScarlet and were dim green (Figure 3B). High-resolution confocal microscopy of live, anesthetized polyps (to prevent them from moving during imaging) revealed various cell types, all derived from the single transplanted i-cell. By morphology, they included the major *Hydractinia* cell lineages: epitheliomuscular cells, neurons, stinging cells (nematocytes), and germ cells (Figures 4 and S6B). Neurons were also identified by anti-RFamide antibodies (Figure 4B). Other cell types, such as gland cells, are difficult to identify *in vivo* within the tissue. Of note, all fluorescent germ cells were oocytes, consistent with the sex of the donor animal. They co-occupied the gonads alongside wild-type sperm from the recipient (Figure 4E). Spawned eggs were fertilized with 291-10 wild-type sperm and became embryos that completed development into larvae (Video S1) and metamorphosed into young, fluorescent colonies, the sexual offspring of the single transplanted i-cell (Figure S6C).

The female clone 106 grows faster than the male clone 291-10 under laboratory conditions. Therefore, over time, the proportion of fluorescent cells in chimeras increased gradually, resulting in

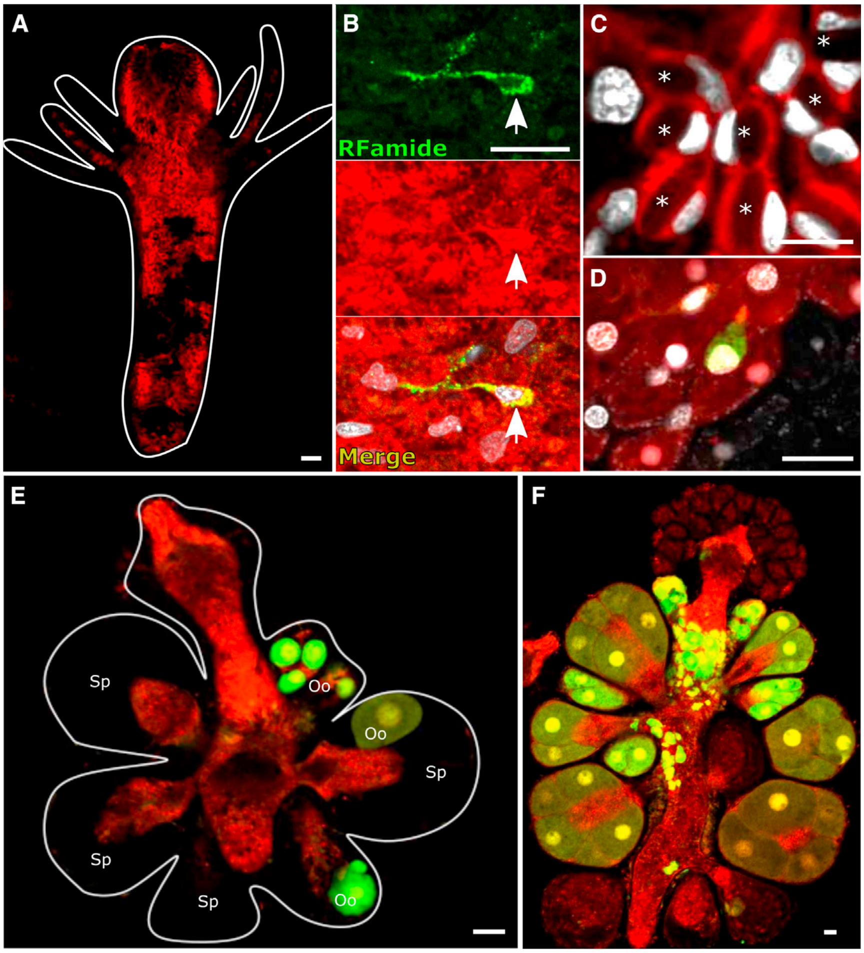

Figure 4. Various donor cell types, derivatives of a single transplanted i-cell, in a chimeric animal

(A) Chimeric feeding polyp. Dark, non-fluorescent areas represent wild-type host tissue.

(B) Donor-derived neuron (labeled by anti-RFamide and anti-mScarlet antibodies) embedded in a sheet of donor epithelial cells.

(C) Nematocytes, each containing nematocyst capsules (asterisks) and a typical crescent-shaped nucleus.

(D) An i-cell (green) lodged between epithelial cells.

(E) Partially taken over sexual polyp, showing donor-derived (green) oocytes in recipient-derived, sperm-filled male sporosac.

(F) A sexual polyp, nearly taken over by donor cells. i-cells and early germ cells are green. Maturing oocytes are also red.

All except (B) are live images. Scale bars, 20 μm.

See also Figure S6 and Video S1.

polyps with varying levels of transgenic, 106 representations (Figure 4). We selectively pruned tissues that contained only a few or no fluorescent cells, thereby tipping the balance in favor of donor cells. Eventually, some polyps in these chimeric colonies became completely red/green with few or no apparent wild-type cells (Figures 5 and 6A). These polyps were isolated and grew into new colonies. Spectral flow cytometry of dissociated polyps originating from 291-10 colonies that had received a single 106 i-cell showed that some of these animals were no longer chimeric, as no wild-type cells were detectable in their tissues, confirming that a total takeover by donor cells had occurred and that all cells of these individuals—both somatic and germ cells—were exclusively derived from the one transplanted i-cell (Figures 6B–6D). The same takeover dynamics occurred in each one of the five chimeras that had received a single donor i-cell. We concluded that the adult population of *Hydractinia* i-cells includes pluripotent stem cells.

DISCUSSION

In asexual planarians, Piwi-1^{high} neoblasts constitute some 40% of the animal's cells.[10] However, only about 15% of them are clonogenic, able to rescue a lethally irradiated worm.[9,10] By contrast, *Hydractinia* Piwi1^{high} i-cells in feeding polyps are relatively rare, comprising only 1% of the animal's cells.[16,24] The observed takeover by all five single-cell grafts suggests that a considerable fraction of i-cells marked by high *Soxb1*, *Piwi1*, and *Piwi2* expression are pluripotent stem cells. Arguably, they represent the most effective stem cell system for long-living clonal animals that generate plant-like colonies without a defined size or shape. By analogy, *Hydractinia* stolons resemble plant stems and shoots, feeding polyps are equivalent to plants' leaves, and sexual polyps fulfill the role of flowers. If i-cells were a mixed population of lineage-restricted stem cells, competition among lineages could result in a local or global shortage of progenitors for certain cell types. A single pluripotent stem cell system ensures that new stolons and polyps (both feeding and sexual) can develop at any region of the colony, provided that at least one i-cell is present at that location.

Given that pluripotency in most animals is limited to a short time frame pre-gastrulation, it would be interesting to speculate about the embryonic origin of i-cells, which are adult pluripotent cells. Like other cnidarians that are diploblastic,[29] *Hydractinia* embryos gastrulate to generate only two germ layers: ectoderm and endoderm.[30] i-cells first appear in the endodermal cell mass but migrate to the epidermis during metamorphosis to become the adult i-cell population.[16] Embryonic i-cells could directly derive from pluripotent blastomeres that do not commit to any germ layer during gastrulation. Alternatively, they could arise from committed endodermal cells, acquiring pluripotency secondarily. Under both scenarios, as i-cells migrate, proliferate, and differentiate into all cell types, eventually all adult tissues will have been derived from i-cells rather than from embryonic ectoderm or endoderm. Therefore, *Hydractinia* embryonic germ layers are merely transient tissues.

The molecular mechanisms that control pluripotency are partly conserved across metazoans.[31–35] This is not surprising,

CellPress
OPEN ACCESS

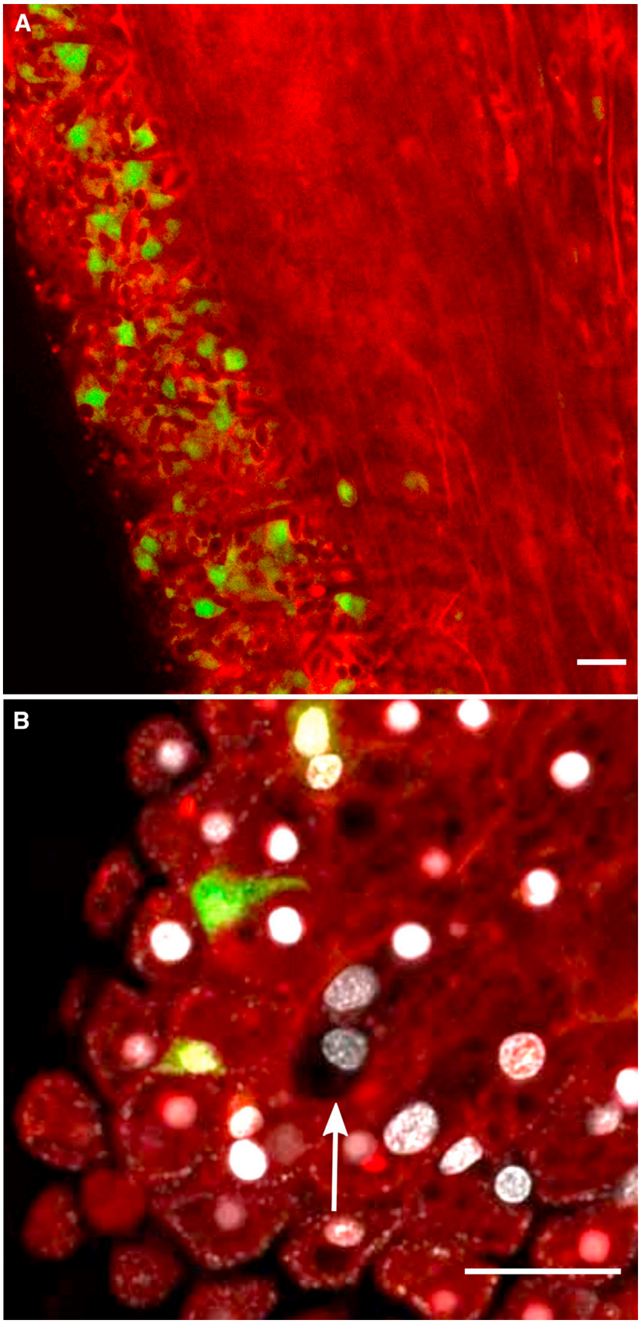

Figure 5. Live imaging of nearly taken over chimera
(A) Longitudinal confocal section through a chimera.
(B) Higher magnification showing a single wild-type epithelial cell embedded in donor-derived tissue (arrow). Differentiated cells are red; i-cells are green. Scale bars, 20 μm.

given that all animals have pluripotent cells in early embryogenesis. However, adult pluripotent stem cells (APSCs) appear to be rare; their appearance is correlated with whole-body regenerative abilities and clonal growth. Mechanistically, APSCs require maintaining or reactivating the embryonic pluripotency gene regulatory network in adulthood. At single-cell resolution, APSCs were shown to exist in only two phyla—Cnidaria and Platyhelminthes[9–11]—they are assumed, though not confirmed, in sponges and acoels.[31,36] Interestingly, colonial tunicates, although clonal and highly regenerative, were suggested to have distinct lineages of adult stem cells, one somatic and one germline.[37] Taken together, although pluripotency per se—being ubiquitous—is probably a primitive metazoan feature, the distribution pattern of APSCs across animal phyla is consistent with two evolutionary scenarios. They could be either a primitive trait, lost by most bilaterians and some cnidarians (e.g., *Hydra*), or a derived feature, evolving multiple times in different lineages.

STAR★METHODS

Detailed methods are provided in the online version of this paper and include the following:

- KEY RESOURCES TABLE
- RESOURCE AVAILABILITY
    - Lead contact
    - Materials Availability
    - Data and code availability
- EXPERIMENTAL MODEL AND SUBJECT DETAILS
    - Animal culture and strains
- METHOD DETAILS
    - Manipulation of Polyps
    - Polyp and Stolon Immunofluorescence Preparation
    - Immunofluorescence
    - Tissue dissociation
    - Generation of cell aggregates
    - Single Cell Transplantation Via Grafting
    - Live Microscopy and Image analysis
    - EdU Staining
    - BrdU Staining
    - Flow Cytometry
- QUANTIFICATION AND STATISTICAL ANALYSIS

SUPPLEMENTAL INFORMATION

Supplemental information can be found online at https://doi.org/10.1016/j.cub.2023.03.039.

ACKNOWLEDGMENTS

We thank Patricia Calcagno, Amy Duclaux, and Laura Ryan for animal culturing and all members of our lab for discussions and advice. Confocal microscopy and flow cytometry were conducted at the Centre for Microscopy and Imaging and at the Flow Cytometry Core Facilities at the University of Galway, respectively. U.F. is a Wellcome Trust Investigator in Science (grant no. 210722/Z/18/Z), and work in the Frank lab was also funded by the NSF EDGE program (grant no. 1923259). G.K. was an Irish Research Council postdoctoral fellow (project ID GOIPD/2020/149). H.R.H. is a doctoral student in the Science Foundation Ireland Centre for Research Training in Genomic Data Science (grant no. 18/CRT/6214).

AUTHOR CONTRIBUTIONS

Á.V. and U.F. conceptualized the study. Á.V. performed experiments. E.T.M. performed EdU/BrdU experiments. Á.V. and G.K. performed aggregate experiments. H.R.H. and Á.V. performed spectral flow cytometry. Á.V., H.R.H., and U.F. wrote the paper. All authors commented on and approved the manuscript.

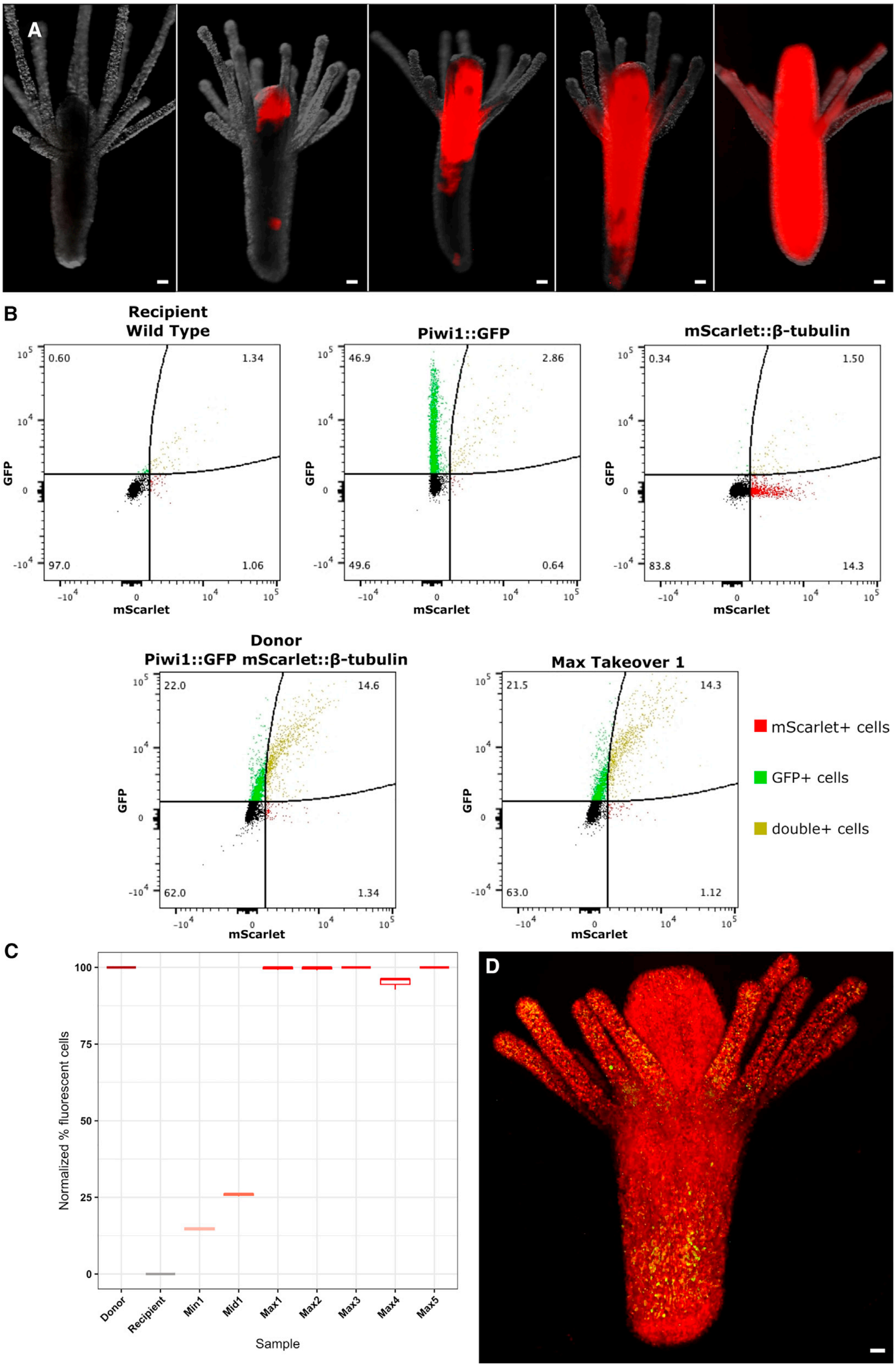

(legend on next page)

CellPress
OPEN ACCESS

DECLARATION OF INTERESTS

The authors declare no competing interests.

Received: December 22, 2022
Revised: February 16, 2023
Accepted: March 13, 2023
Published: April 6, 2023

REFERENCES

1. Hanna, J.H., Saha, K., and Jaenisch, R. (2010). Pluripotency and cellular reprogramming: facts, hypotheses, unresolved issues. Cell *143*, 508–525.
2. Yilmaz, A., and Benvenisty, N. (2019). Defining human pluripotency. Cell Stem Cell *25*, 9–22. https://doi.org/10.1016/j.stem.2019.06.010.
3. Rinkevich, Y., Lindau, P., Ueno, H., Longaker, M.T., and Weissman, I.L. (2011). Germ-layer and lineage-restricted stem/progenitors regenerate the mouse digit tip. Nature *476*, 409–413. https://doi.org/10.1038/nature10346.
4. Kragl, M., Knapp, D., Nacu, E., Khattak, S., Maden, M., Epperlein, H.H., and Tanaka, E.M. (2009). Cells keep a memory of their tissue origin during axolotl limb regeneration. Nature *460*, 60–65. https://doi.org/10.1038/nature08152.
5. Martínez, D.E. (1998). Mortality patterns suggest lack of senescence in hydra. Exp. Gerontol. *33*, 217–225.
6. Sahu, S., Dattani, A., and Aboobaker, A.A. (2017). Secrets from immortal worms: what can we learn about biological ageing from the planarian model system? Semin. Cell Dev. Biol. *70*, 108–121. https://doi.org/10.1016/j.semcdb.2017.08.028.
7. Khan, U.W., and Newmark, P.A. (2022). Somatic regulation of female germ cell regeneration and development in planarians. Cell Rep. *38*, 110525. https://doi.org/10.1016/j.celrep.2022.110525.
8. Issigonis, M., Redkar, A.B., Rozario, T., Khan, U.W., Mejia-Sanchez, R., Lapan, S.W., Reddien, P.W., and Newmark, P.A. (2022). A Krüppel-like factor is required for development and regeneration of germline and yolk cells from somatic stem cells in planarians. PLOS Biol. *20*, e3001472, https://doi.org/10.1371/journal.pbio.3001472.
9. Wagner, D.E., Wang, I.E., and Reddien, P.W. (2011). Clonogenic neoblasts are pluripotent adult stem cells that underlie planarian regeneration. Science *332*, 811–816. https://doi.org/10.1126/science.1203983.
10. Zeng, A., Li, H., Guo, L., Gao, X., McKinney, S., Wang, Y., Yu, Z., Park, J., Semerad, C., Ross, E., et al. (2018). Prospectively isolated tetraspanin+ neoblasts are adult pluripotent stem cells underlying planaria regeneration. Cell *173*, 1593–1608.e20. https://doi.org/10.1016/j.cell.2018.05.006.
11. Bosch, T.C.G., and David, C.N. (1987). Stem cells of *Hydra magnipapillata* can differentiate into somatic cells and germ line cells. Dev. Biol. *121*, 182–191.
12. Bode, H.R. (1996). The interstitial cell lineage of hydra: a stem cell system that arose early in evolution. J. Cell Sci. *109*, 1155–1164.
13. Juliano, C.E., Reich, A., Liu, N., Götzfried, J., Zhong, M., Uman, S., Reenan, R.A., Wessel, G.M., Steele, R.E., and Lin, H. (2014). PIWI proteins and PIWI-interacting RNAs function in Hydra somatic stem cells. Proc. Natl. Acad. Sci. USA *111*, 337–342. https://doi.org/10.1073/pnas.1320965111.
14. Siebert, S., Farrell, J.A., Cazet, J.F., Abeykoon, Y., Primack, A.S., Schnitzler, C.E., and Juliano, C.E. (2019). Stem cell differentiation trajectories in Hydra resolved at single-cell resolution. Science *365*, eaav9314, https://doi.org/10.1126/science.aav9314.
15. Leclère, L., Jager, M., Barreau, C., Chang, P., Le Guyader, H., Manuel, M., and Houliston, E. (2012). Maternally localized germ plasm mRNAs and germ cell/stem cell formation in the cnidarian Clytia. Dev. Biol. *364*, 236–248. https://doi.org/10.1016/j.ydbio.2012.01.018.
16. Chrysostomou, E., Flici, H., Gornik, S.G., Salinas-Saavedra, M., Gahan, J.M., McMahon, E.T., Thompson, K., Hanley, S., Kilcoyne, M., Schnitzler, C.E., et al. (2022). A cellular and molecular analysis of SoxB-driven neurogenesis in a cnidarian. eLife *11*, e78793, https://doi.org/10.7554/eLife.78793.
17. Rosengarten, R.D., and Nicotra, M.L. (2011). Model systems of invertebrate allorecognition. Curr. Biol. *21*, R82–R92.
18. Gahan, J.M., Bradshaw, B., Flici, H., and Frank, U. (2016). The interstitial stem cells in *Hydractinia* and their role in regeneration. Curr. Opin. Genet. Dev. *40*, 65–73. https://doi.org/10.1016/j.gde.2016.06.006.
19. Bradshaw, B., Thompson, K., and Frank, U. (2015). Distinct mechanisms underlie oral vs aboral regeneration in the cnidarian *Hydractinia echinata*. eLife *4*, e05506. https://doi.org/10.7554/eLife.05506.
20. Weismann, A. (1883). Die Entstehung der Sexualzellen bei Hydromedusen (Gustav Fischer).
21. Müller, W.A. (1964). Experimentele Untersuchungen über Stockentwicklung, Polypendifferenzierung und Sexualchimären bei *Hydractinia echinata*. Roux' Arch. für Entwicklungsmechanik *155*, 181–268.
22. Müller, W.A., Teo, R., and Frank, U. (2004). Totipotent migratory stem cells in a hydroid. Dev. Biol. *275*, 215–224.
23. Künzel, T., Heiermann, R., Frank, U., Müller, W.A., Tilmann, W., Bause, M., Nonn, A., Helling, M., Schwarz, R.S., and Plickert, G. (2010). Migration and differentiation potential of stem cells in the cnidarian *Hydractinia* analysed in GFP-transgenic animals and chimeras. Dev. Biol. *348*, 120–129.
24. DuBuc, T.Q., Schnitzler, C.E., Chrysostomou, E., McMahon, E.T., Febrimarsa, G., Gahan, J.M., Buggie, T., Gornik, S.G., Hanley, S., Barreira, S.N., et al. (2020). Transcription factor AP2 controls cnidarian germ cell induction. Science *367*, 757–762. https://doi.org/10.1126/science.aay6782.
25. Chen, R., Sanders, S.M., Ma, Z., Paschall, J., Chang, E.S., Riscoe, B.M., Schnitzler, C.E., Baxevanis, A.D., and Nicotra, M.L. (2022). XY sex determination in a cnidarian. https://doi.org/10.1101/2022.03.22.485406.
26. Mali, B., Millane, R.C., Plickert, G., Frohme, M., and Frank, U. (2011). A polymorphic, thrombospondin domain-containing lectin is an oocyte marker in *Hydractinia*: implications for germ cell specification and sex determination. Int. J. Dev. Biol. *55*, 103–108.
27. Cadavid, L.F., Powell, A.E., Nicotra, M.L., Moreno, M., and Buss, L.W. (2004). An invertebrate histocompatibility complex. Genetics *167*, 357–365.
28. Nicotra, M.L. (2022). The Hydractinia allorecognition system. Immunogenetics *74*, 27–34. https://doi.org/10.1007/s00251-021-01233-6.
29. Technau, U. (2020). Gastrulation and germ layer formation in the sea anemone Nematostella vectensis and other cnidarians. Mech. Dev. *163*, 103628. https://doi.org/10.1016/j.mod.2020.103628.
30. Kraus, Y., Flici, H., Hensel, K., Plickert, G., Leitz, T., and Frank, U. (2014). The embryonic development of the cnidarian Hydractinia echinata. Evol. Dev. *16*, 323–338. https://doi.org/10.1111/ede.12100.

Figure 6. Analysis of the takeover dynamics by donor i-cell

(A) Live imaging of various degrees of chimerism, from a wild-type polyp (left) to apparently a completely taken over one (right).

(B) Spectral flow cytometry of wild-type polyps (clone 291-10), Piwi1:GFP animals (clone 107), β-tubulin::mScarlet animal (clone 102), donor (clone 106), and a taken over polyp.

(C) Percentages of transgenic fluorescent cells across three technical replicates in animals that have received a single donor i-cell, normalized to donor (clone 106) and recipient (clone 291-10). Various degrees of chimerism, from low to complete takeover, are shown.

(D) Maximum projection of a confocal live imaging stack of an apparent taken over polyp, showing i-cells in green and differentiated somatic cells in red. Scale bars, 40 μm.

31. Alié, A., Hayashi, T., Sugimura, I., Manuel, M., Sugano, W., Mano, A., Satoh, N., Agata, K., and Funayama, N. (2015). The ancestral gene repertoire of animal stem cells. Proc. Natl. Acad. Sci. USA *112*, E7093–E7100. https://doi.org/10.1073/pnas.1514789112.

32. Alié, A., Leclère, L., Jager, M., Dayraud, C., Chang, P., Le Guyader, H., Quéinnec, E., and Manuel, M. (2011). Somatic stem cells express Piwi and Vasa genes in an adult ctenophore: ancient association of "germline genes" with stemness. Dev. Biol. *350*, 183–197. https://doi.org/10.1016/j.ydbio.2010.10.019.

33. Juliano, C.E., Swartz, S.Z., and Wessel, G.M. (2010). A conserved germline multipotency program. Development *137*, 4113–4126. https://doi.org/10.1242/dev.047969.

34. Wagner, D.E., Ho, J.J., and Reddien, P.W. (2012). Genetic regulators of a pluripotent adult stem cell system in planarians identified by RNAi and clonal analysis. Cell Stem Cell *10*, 299–311. https://doi.org/10.1016/j.stem.2012.01.016.

35. Önal, P., Grün, D., Adamidi, C., Rybak, A., Solana, J., Mastrobuoni, G., Wang, Y., Rahn, H.P., Chen, W., Kempa, S., et al. (2012). Gene expression of pluripotency determinants is conserved between mammalian and planarian stem cells. EMBO J. *31*, 2755–2769. https://doi.org/10.1038/emboj.2012.110.

36. Kimura, J.O., Bolaños, D.M., Ricci, L., and Srivastava, M. (2022). Embryonic origins of adult pluripotent stem cells. Cell *185*, 4756–4769.e13. https://doi.org/10.1016/j.cell.2022.11.008.

37. Laird, D.J., De Tomaso, A.W., and Weissman, I.L. (2005). Stem cells are units of natural selection in a colonial ascidian. Cell *123*, 1351–1360.

CellPress
OPEN ACCESS

STAR★METHODS

KEY RESOURCES TABLE

REAGENT or RESOURCE	SOURCE	IDENTIFIER
Antibodies		
Anti-BrdU	Sigma	CAT#B5002
Guinea Pig Polyclonal Anti-GFP	Synaptic Systems	CAT#132005
Rabbit Polyclonal Anti-Piwi1	In house	DuBuc et al.[24]
Chicken Polyclonal Anti-mScarlet	Synaptic Systems	CAT#409 006
Rabbit Polyclonal Anti-RFamide	Gunther Plickert	N/A
Deposited data		
Raw Data for flow cytometry	FigShare	https://doi.org/10.6084/m9.figshare.22232173
Experimental models: Organisms/strains		
Wild type *Hydractinia symbiolongicarpus* male	In house	291-10
Transgenic *Hydractinia* clone Piwi1::GFP β-tubulin::mScarlet; female	DuBuc et al.[24]	106
Transgenic *Hydractinia* clone β-tubulin::mScarlet; male	DuBuc et al.[24]	102
Transgenic *Hydractinia* clone Piwi1::GFP; female	DuBuc et al.[24]	107, 123
Software and algorithms		
ImageJ	https://imagej.net/ij/download/src/	Version 2.8.0
SpectroFlo	https://cytekbio.com/pages/spectro-flo	Version 3.0
ImageJ	https://imagej.net/ij/download/src/	Version 2.8.0

RESOURCE AVAILABILITY

Lead contact

Requests for additional information or for resources and reagents should be sent to the lead contact, Uri Frank (uri.frank@universityofgalway.ie).

Materials Availability

This study did not generate new unique reagents.

Data and code availability

- Raw data have been deposited at Figshare depository and are publicly available as of the date of publication. DOIs are listed in the key resources table.
- This paper does not report original code.
- Further information for reanalyzing of data is available from the lead contact upon request.

EXPERIMENTAL MODEL AND SUBJECT DETAILS

Animal culture and strains

Adult *Hydractinia symbiolongicarpus* clones were cultured in artificial seawater (salinity 28-32 ppt) at 20-22°c under a 14:10 light:dark regime. Animals were grown on glass microscope slides in 3D printed racks. They were fed four times a week with hatched *Artemia franciscana* and once per week with ground oyster.

Spawning of gametes occurs approximately 90 minutes post-light exposure. Fertilized embryos were allowed to develop at RT. Once they reached three days old and formed free-swimming planula, they are induced to metamorphose by incubating the larvae in a 4:1 solution of filtered sea water (FSW) and 580 mM CsCl for three hours. This is done to mimic the process that occurs naturally in the wild on the hermit crab shell due to the presence of a bacterial film. These larvae are then washed in FSW and settled on glass slides to complete metamorphosis.

The following animal strains (clones) were used: 291-10 is a wild type male; clone 107 and 123 are Piwi1::GFP reporter animals, expressing GFP only in i-cells and germ cells; 102 is a male β-tubulin::mScarlet reporter, expressing mScarlet in all cells, except for i-cells; 106 is female, double transgenic animal, expressing GFP only in i-cells and germ cells, and mScarlet only in all other cells.

METHOD DETAILS

Manipulation of Polyps

Hydractinia colonies were placed in 4% $MgCl_2$ for 10 minutes to anesthetize the animal and limit movement. Individual sexual and feeding polyps were extracted from the colony by cutting horizontally across the base of the polyp, closest to the stolon. The cut polyps were then transferred to FSW. For re-settling of colonies, a stolon with a single feeding polyp was cut from the colony. It was then placed on a new slide where the stolon attached and resumed growth to become a new colony.

Polyp and Stolon Immunofluorescence Preparation

For immunofluorescence of polyps with extended stolon, *Hydractinia* colonies were placed in 4% $MgCl_2$ for 10 minutes to anaesthetize the animal. Individual feeding polyps were cut and placed into a glass dish in FSW. They were rocked for 7-9 days and fed every day until they had grown long stolon that were then used in immunofluorescence experiments.

Immunofluorescence

Immunofluorescence (IF) was performed as previously described.[24] Animals were anesthetized in 4% $MgCl_2$ for 10 minutes then fixed in 4% PFA in FSW overnight at 4°C. Samples were then washed three times with PBS with 0.5% Triton (PBSTx). For long-term storage, the tissue was dehydrated by 5-minute washes with increasing ethanol concentrations diluted in PBSTx (25%, 50%, 75% and 100% ethanol) and stored at -20°C. The dehydrated tissue was then gradually rehydrated by 10-minute washes in decreasing concentrations of ethanol in PBSTx. Samples were then washed three times in PBST and incubated for 1 hour in filtered 3% BSA in PBSTx. Primary antibodies incubation was performed overnight at 4°C. Next, samples were washed with PBST three times for 10 minutes each at RT while being rocked. They were then blocked again in 3% BSA/ PBSTx/5% goat serum for 15 minutes at RT. The secondary antibody was then added and incubated for 60 minutes at RT. Samples were washed in PBSTx three times, incubated in Hoechst 33258 (1 in 1000; stock: 20mg/ml; Sigma-Aldrich; B2883) for 30-60 minutes, and mounted in TDE on glass microscopy slides and covered with a glass coverslip that was then sealed with clear nail polish.

Tissue dissociation

Adult polyps were incubated in calcium- and magnesium-free seawater (CMFSW) for 8.5 minutes at RT while rocking. The CMFSW was then removed and replaced with 200 μL filtered seawater (FSW). The polyps were then immediately dissociated by drawing them in and out of a 25 G needle until all clumps disappeared. The cell suspension was then passed through a 100 μm and then a 40 μm filter.

Generation of cell aggregates

To generate cell aggregates, a filtered cell suspension was placed in a 200 μL PCR tube and centrifuged at 800 x g for 90 minutes until cells were pelleted and aggregates formed. The aggregates were dislodged from the base of the PCR tube and placed in a petri dish of FSW, placed on a rocker at RT for 3-5 days until a new polyp regenerated from the aggregate. Cell suspension from a minimum number of 25 adult polyps was necessary to generate an aggregate that that could regenerate a single polyp, suggesting that many cells are lost in the process.

Single Cell Transplantation Via Grafting

The donor animal (Piwi1::GFP β-tubulin::mScarlet; female 106) and the recipient animal (wild type male 291-10) were settled on slides in close proximity to one another. They were allowed to grow until stolonal contact was established. The recipient wild type stolons were observed using fluorescence stereomicroscopy to follow single i-cells migrating from the donor into the recipient stolon. Once this event occurred, the piece of recipient tissue containing a single donor i-cell was cut away from the rest of the stolon. Most of the recipient tissue was also removed to leave a small piece of stolon. This piece of stolon was cultured and monitored under a fluorescence stereomicroscope to observe the progeny of the initial transplanted i-cell. Images were taken regularly to observe the increase and distribution of the single i-cell's progeny. To ensure enrichment of the donor cells progeny, large sections of wild type tissue were periodically removed from the chimera.

Live Microscopy and Image analysis

Live animals were stained with Hoechst 33342 for 30 minutes and were then placed in $MgCl_2$ to anesthetize the animals to limit movement. They were then placed on a slide and covered with a coverslip. Live images were taken using a confocal microscope (Olympus FV3000) With high magnification of 20x and 60x to identify cell types by morphology and fluorescent protein expression.

EdU Staining

EdU staining was carried out with Click-iT EdU Alexa Fluor 594 HCS Assay (Invitorgen, cat no. C10339). Fixation of animals was performed in 4% PFA/PBS for 30 minutes at RT. Samples were washed twice in 0.5% PBS Triton (PBSTx) for 20 minutes. The PBSTx solution was removed and replaced with 3% BSA, 0.5% PBSTx solution. During this time, the Click-iT reagents were prepared in accordance with the manufacturer's protocol. The BSA solution was removed and the Click-iT reaction cocktail was added to the tissue and incubated for 1 hour in the dark. The reaction cocktail was then removed, and the tissue was incubated in 3% BSA/ 0.5% PBSTx for five minutes for five times. Standard DNA labelling or IF staining of samples were then carried out as per their respective protocols.

BrdU Staining

Fixation of animals was performed in 4% PFA/PBS for 30 minutes at RT. The tissue was washed 3 times in 0.5% PBSTx for 5 minutes per wash. Samples were treated with 2 M HCl in PBS for 30 minutes. They were then washed three times in 0.5% PBSTx for 5 minutes. Samples were then placed in 3% BSA, 0.5% PBSTx solution for one hour. The anti-BrdU primary antibody (Sigma: B5002) (in a dilution of 1/500 in 3% BSA) was added to the tissue and incubated overnight at 4°C. The next day, the tissue was washed 3 times in 0.5% PBSTx for 5 minutes. Samples were then incubated in 0.5% PBSTx/0.3% BSA/5% goat serum for 60 minutes. Secondary antibody was added to the solution and incubated for 60 minutes at RT in the dark. Samples were then washed 3 times in 0.5% PBSTx for 5 minutes per time and incubated in Hoechst 33258 (use: 1 in 1000; stock: 20mg/ml; Sigma-Aldrich; B2883) for 30-60 minutes.

Flow Cytometry

Whole feeding polyp tissue samples were dissociated in 10 μL FSW per polyp as per dissociation protocol. Nuclear staining of the resultant single cell suspensions was performed by incubation with 37.5 μg/mL Hoechst 33342 (Sigma Cat #14533) for 20 min at RT.

A Cytek Northern Lights NL-3000 spectral flow cytometer (Cytekbio, NL), calibrated and quality controlled per manufacturers guidelines, was used. Data analysis was performed using Cytek's SpectroFlo and FlowJo. Full spectrum signatures were obtained both for unstained and for Hoechst stained wild type cells (Clone 291), from the single transgenic Piwi1::GFP polyps (clone 107) and from single transgenic β-tubulin::mScarlet polyps (clone 102) to allow spectral unmixing and subtraction of autofluorescence. Following this, all Hoechst-stained controls were analysed. Single, nucleated cells were gated based on Hoechst-staining (positive vs negative for Hoechst emission spectra) and size (FSC-H vs FSC-A). Subsequently, single positive cells for GFP (GFP^+), mScarlet ($mScarlet^+$), and double positive cells for both GFP and mScarlet ($double^+$) were gated as subpopulations, based on GFP^+ vs $mScarlet^+$ in the single stained controls. Hoechst stained double transgenic (clone 106) and Hoechst stained recipient samples were analysed based on this gating strategy and the proportion of cells within the combined GFP^+ or $mScarlet^+$ or $double^+$ gate was obtained. For the purpose of displaying the data, all plots show down sampled data based on the gate for single, nucleated cells. To account for background the proportion of cells falling into the combined gate in the Hoechst wildtype control was subtracted from all samples and controls. The proportion of cells in the combined gate in the samples were then normalised against the proportion of cells in the combined gate in double transgenic donor (clone 106) control (by considering the control to be 100% fluorescent transgenic). This resulted in a percentage of fluorescent transgenic cells per sample which was comparable to both the donor (clone 106) and the recipient (clone 291-10). These percentages were then plotted using ggplot2 in R, with three technical replicates per sample shown.

QUANTIFICATION AND STATISTICAL ANALYSIS

Flow cytometry data analysis was performed using Cytek's SpectroFlo and FlowJo. Plots were made using ggplot2 in R.

Current Biology, Volume *33*

Supplemental Information

Pluripotent, germ cell competent adult stem cells underlie cnidarian regenerative ability and clonal growth

Áine Varley, Helen R. Horkan, Emma T. McMahon, Gabriel Krasovec, and Uri Frank

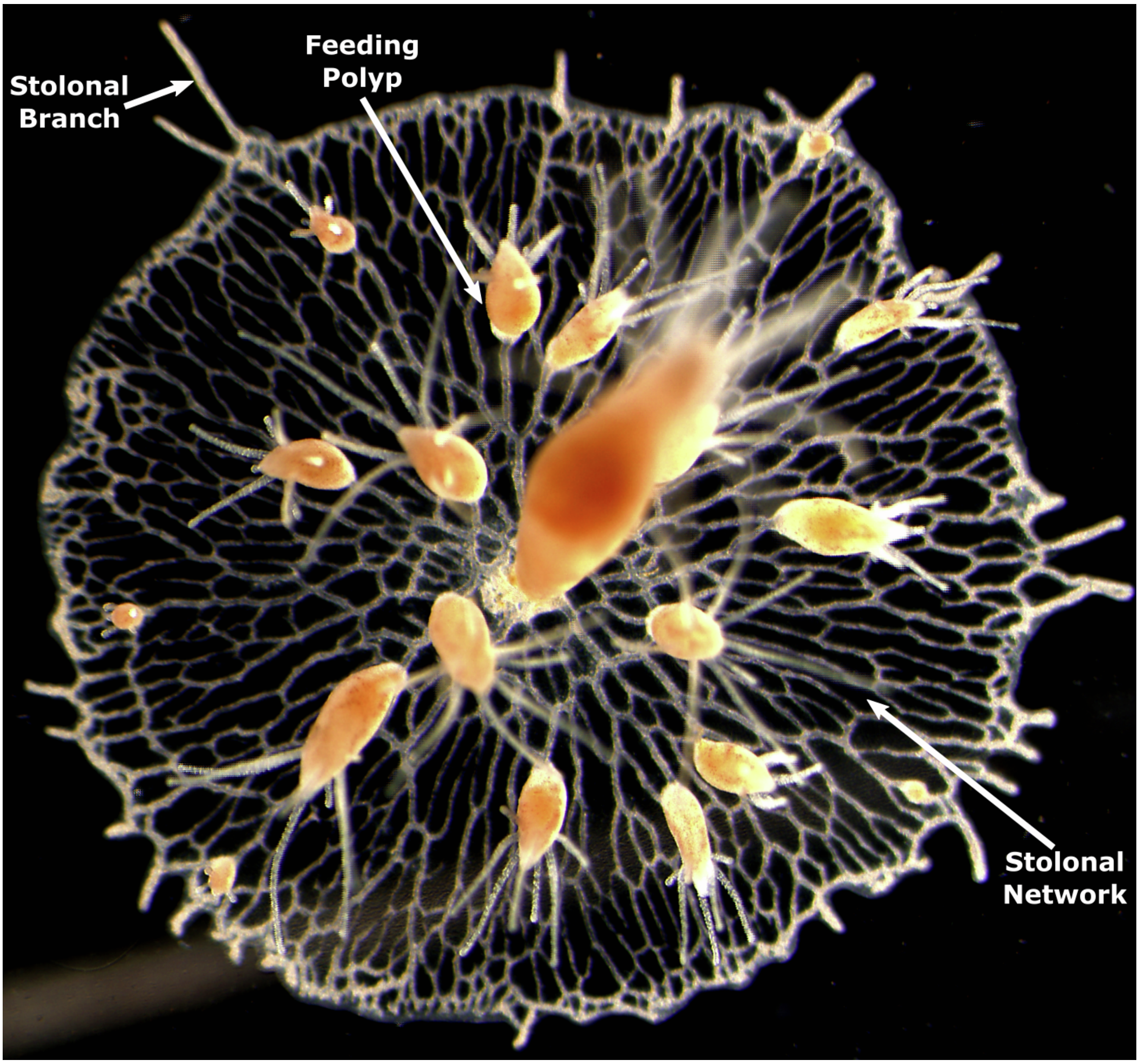

Figure S1. **Feeding polyps and stolonal network of a young colony. Related to Figure 1.**

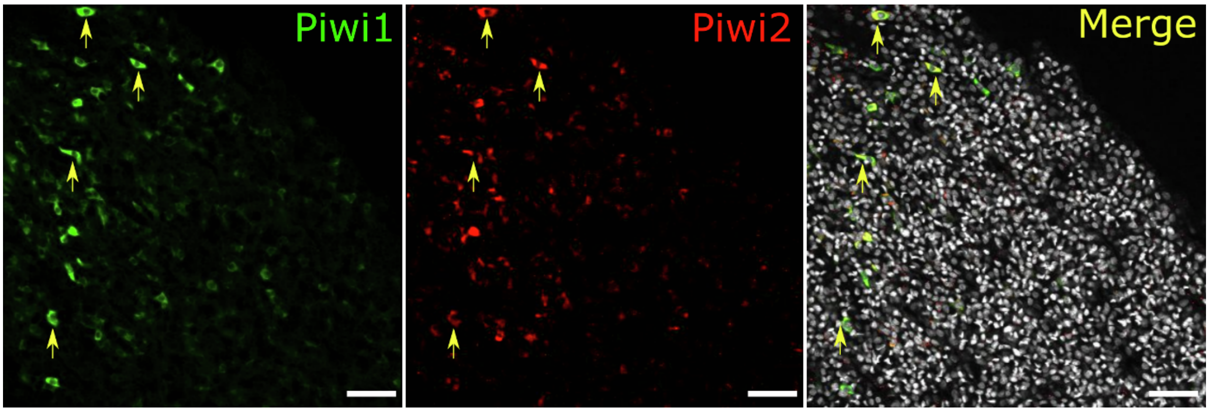

Figure S2. **Piwi1 and Piwi2 double labeling of i-cells. Related to Figure 2.**

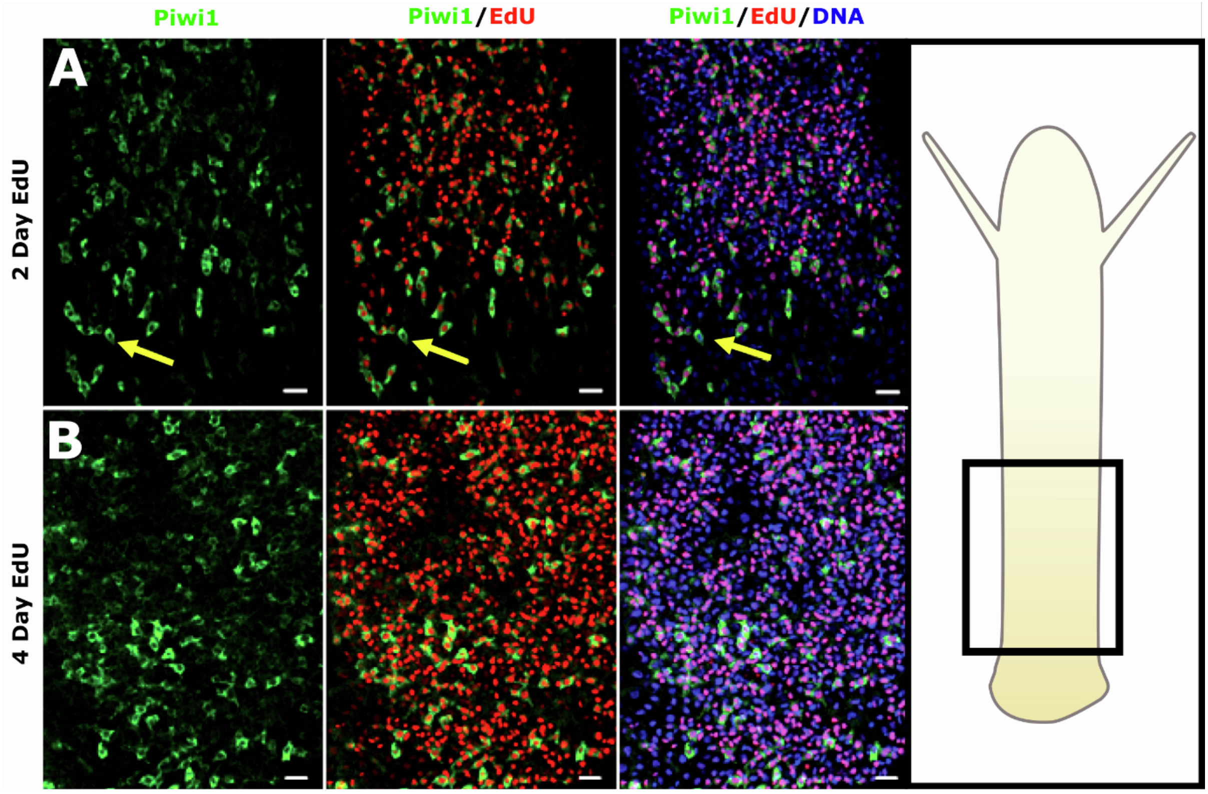

Figure S3. **Fast cycling i-cells in polyps. Related to Figure 2.** (A) EdU incorporation in feeding polyps after 2 days. (B) EdU incorporation after 4 days. Nearly all i-cells have gone through S-phase and are EdU$^+$. Arrow indicates EdU negative i-cell. Scale bars 20 μm.

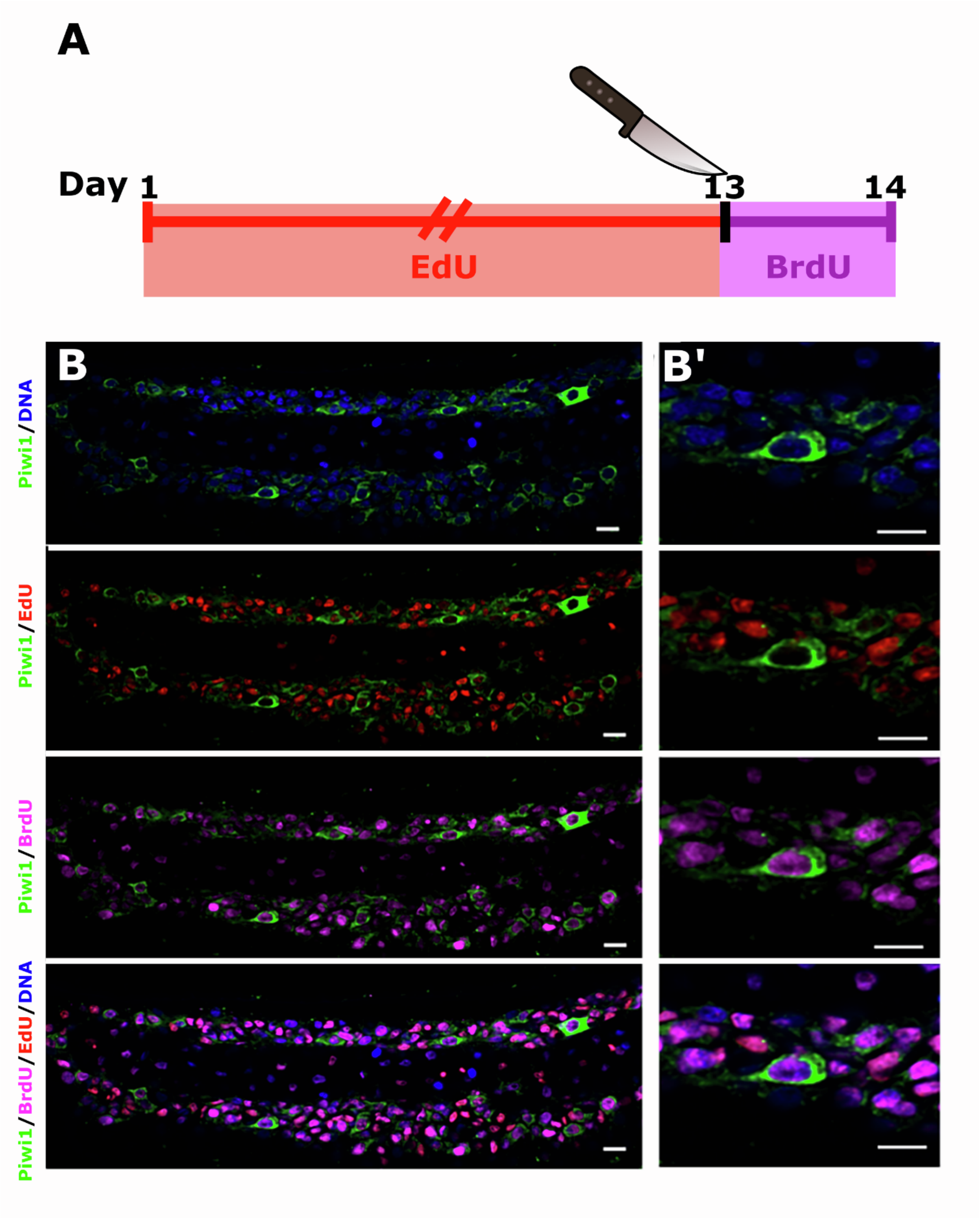

Figure S4. **Slow cycling i-cells in stolons. Related to Figure 2.** (A) Animals were incubated in EdU for 12 days, injured, and incubated in BrdU for 24 hours. (B) i-cells that had been quiescent for at least 12 days but reentered the cell cycle following injury are shown. (B') Higher magnification of a single i-cell. Scale bars 10 µm.

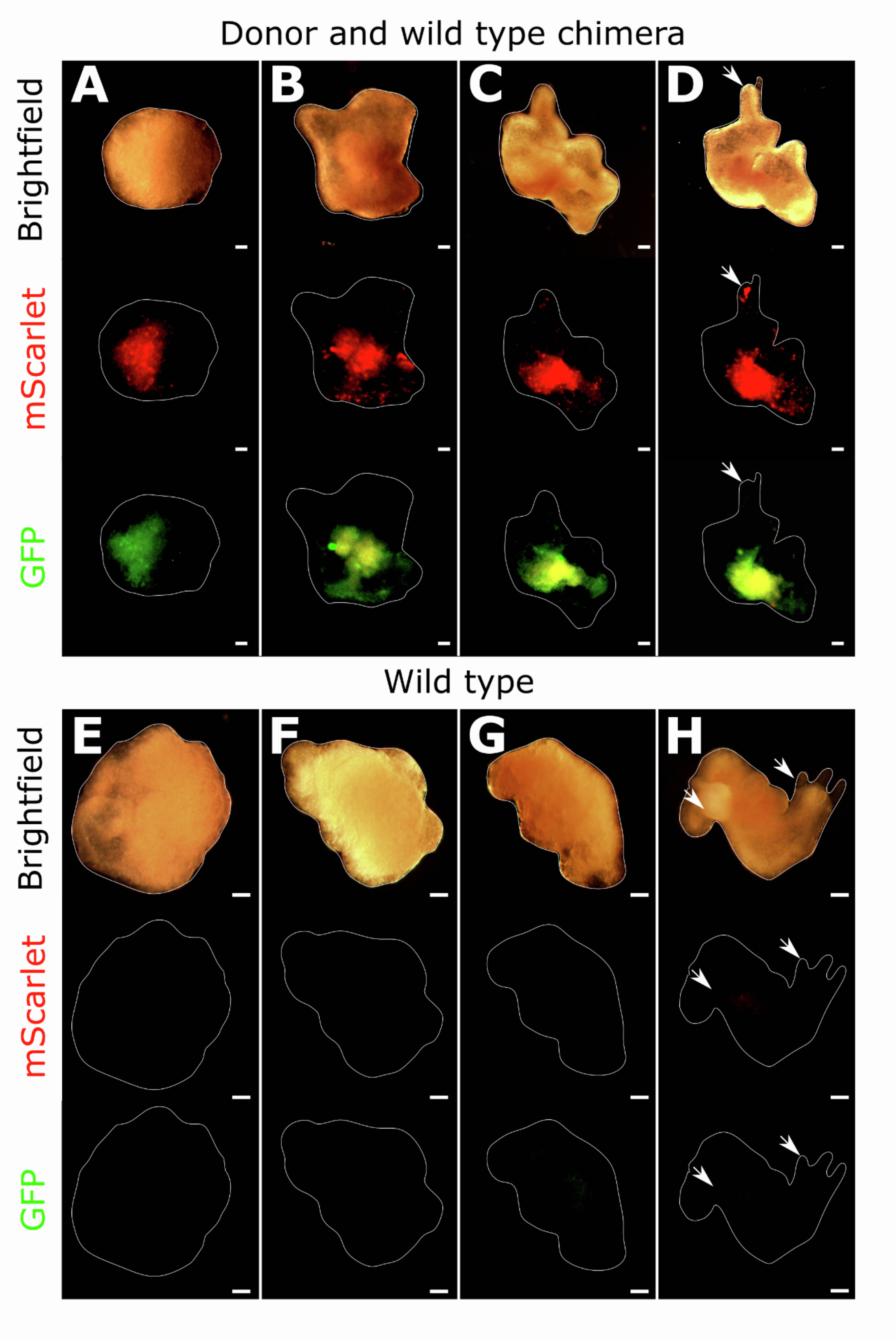

Figure S5. **Live imaging of reaggregated dissociated cells. Related to Figure 3.** (A-D) Mixed cell aggregate composed of dissociated 291-10 and 106 polyps. (E-H) Wild type (291-10) aggregates. (A&E) Day 1. (B&F) Day 3 (C&G) Day 5. (D&H) Day 7. Arrows denote regenerating polyps. Scale bars 20 µm.

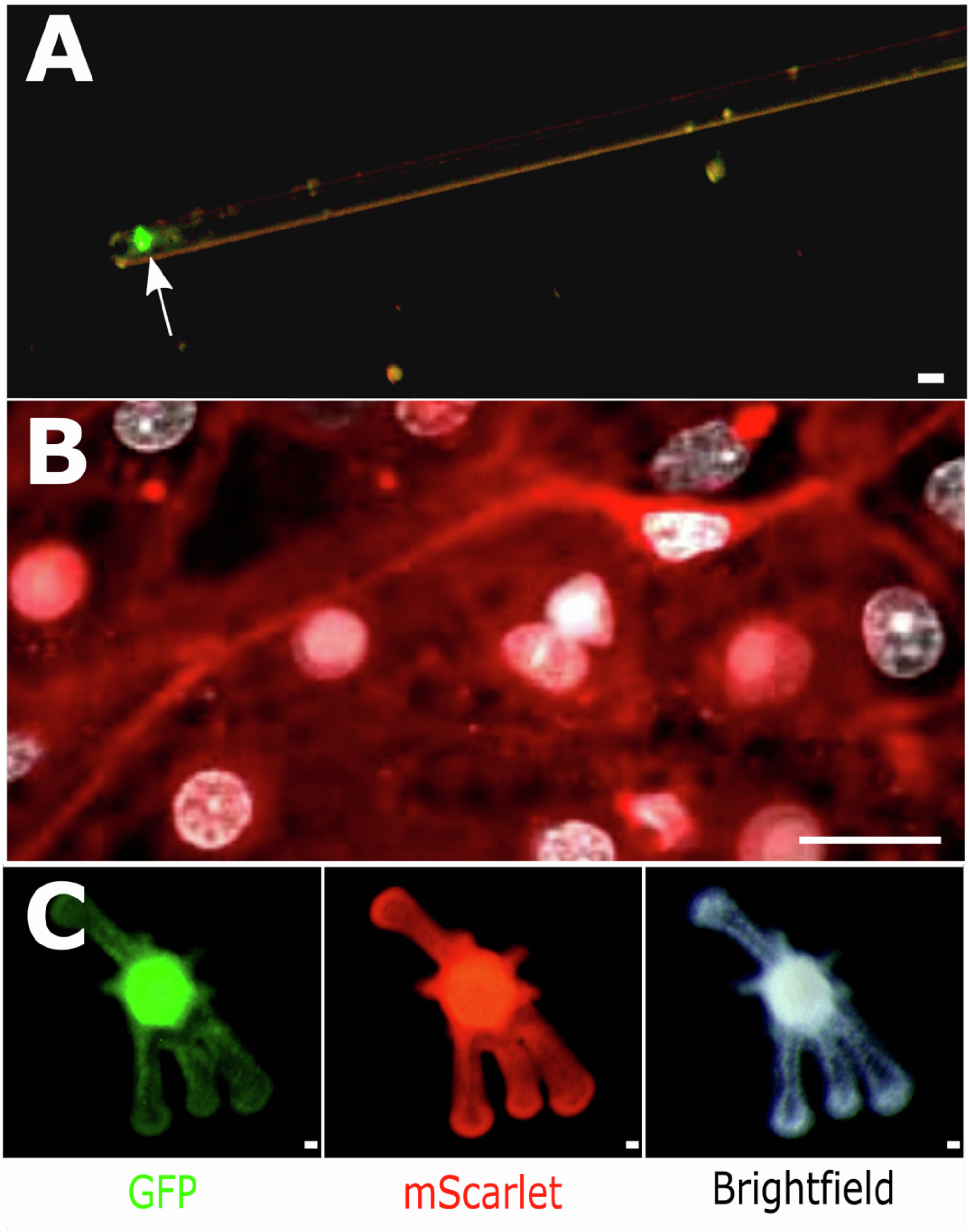

Figure S6. **Isolated transgenic i-cell, donor-derived neuron, and second-generation transgenic colony. Related to Figure 4.** (A) single transgenic i-cell, aspirated in a micropipette from a dissociated 106 polyp. (B) Live image of mScarlet-positive, donor-derived neuron embedded in a chimeric recipient's tissue. (DNA is shown in grey. (C) Young transgenic colony, derived from a single transplanted i-cell. Scale bars 10 µm in A and 20 µm in B & C.
